# Association of LBX1 Gene Methylation Level with Disease Severity in Patients with Idiopathic Scoliosis: Study on Deep Paravertebral Muscles

**DOI:** 10.3390/genes13091556

**Published:** 2022-08-29

**Authors:** Piotr Janusz, Małgorzata Tokłowicz, Mirosław Andrusiewicz, Małgorzata Kotwicka, Tomasz Kotwicki

**Affiliations:** 1 Department of Spine Disorders and Pediatric Orthopedics, Poznan University of Medical Sciences, 28 Czerwca 1956 r. Street 135/147, 61-545 Poznań, Poland; 2 Chair and Department of Cell Biology, Poznan University of Medical Sciences, Rokietnicka 5D, 60-806 Poznań, Poland

**Keywords:** idiopathic scoliosis, scoliosis progression, DNA methylation, ladybird homeobox 1 gene (*LBX1*), pyrosequencing

## Abstract

Idiopathic scoliosis (IS) is a multifactorial disease with a genetic background. The association of Ladybird Homeobox 1 (*LBX1*) polymorphisms with IS has been proven in multiple studies. However, the epigenetic mechanisms have not been evaluated. This study aimed to evaluate the *LBX1* methylation level in deep paravertebral muscles in order to analyze its association with IS occurrence and/or IS severity. Fifty-seven IS patients and twenty non-IS patients were examined for the paravertebral muscles’ methylation level of the *LBX1* promoter region. There was no significant difference in methylation level within paravertebral muscles between patients vs. controls, except for one CpG site. The comparison of the paravertebral muscles’ *LBX1* promoter region methylation level between patients with a major curve angle of ≤70° vs. >70° revealed significantly higher methylation levels in 17 of 23 analyzed CpG sequences at the convex side of the curvature in patients with a major curve angle of >70° for the reverse strand promoter region. The association between *LBX1* promoter methylation and IS severity was demonstrated. In patients with severe IS, the deep paravertebral muscles show an asymmetric *LBX1* promoter region methylation level, higher at the convex scoliosis side, which reveals the role of locally acting factors in IS progression.

## 1. Introduction

Idiopathic scoliosis (IS) is the most common structural deformity of the spine in adolescents. The curvature may remain stable or progress to severe deformation [1]. It is associated with back pain and cosmetic and psychological burdens [2]. Severe IS can lead to pulmonary impairment and functional disability [3]. IS is a multifactorial disease with an important genetic background, probably modulated by environmental factors, which are claimed to impact IS occurrence or progression [4]. Although many genetic studies concerning the IS genetic background have been conducted and several target genes suggested, most of them have not been confirmed in replication studies [5,6,7,8].

Although the *LBX1* (Ladybird Homeobox 1) association with IS has been established using genome-wide association studies [9,10,11], little is known in general about LBX1 function in humans and specifically about its mechanism in IS onset and progression [12,13]. It is known that in vertebrates, the *LBX1* gene plays an essential role in regulating muscle precursor cell migration, maintaining their migratory potential, and promoting muscle precursor cell proliferation [14]. It is also important in specifying dorsal spinal cord interneurons and neural tube closure [15,16]. The molecular mechanisms by which *LBX1* contributes to the development and propagation of neurons need to be explored further in muscle and other tissues [13]. Although *LBX1* expression in developing skeletal muscles and the nervous system during embryogenesis has been described [12,13], the epigenetic impact on paravertebral muscle tissue and its role in IS development is unknown.

According to Grauers et al., the risk of developing scoliosis in a particular subject depends on an additive effect of genetic factors and the impact of environmental factors [17]. Many environmental factors have been evaluated in search of an association with IS. Among the best proven can be listed: low D vitamin level [18], high selenium level [19], and low BMI [20]. It is difficult to indicate a prevalent theory. Thus, IS is considered a multifactorial disease. The higher prevalence of scoliosis in families with an affected member compared to the general population supports the importance of the idea of genetic background. It is a well-described phenomenon associated with the degree of relationship [20,21,22]. However, twin studies show that genetic impact is limited, and other factors are very important [17]. Crosstalk between already described factors and searching for new ones seems to be essential for understanding the reasons for the occurrence and progression of IS [23,24].

It is suggested that epigenetic factors may represent a linkage between the genome and the environment for IS [25,26]. DNA methylation is an epigenetic DNA modification associated with the regulatory regions of many genes. It may alter genes’ expression but does not change the DNA sequence. Genomic DNA methylation occurs mostly on cytosines that precede a guanine nucleotide called CpG sites. When located in a gene promoter, DNA methylation typically acts to repress gene expression [27]. There have been described thousands of genes that exhibit DNA methylation differences between, e.g., males and females in human skeletal muscle. It may modulate mechanisms controlling muscle metabolism and health [28], as well as muscle development [29].

Few studies describing the role of DNA methylation in IS have been published [26,30,31,32,33]. However, epigenetic mechanisms have not been evaluated for the *LBX1* gene yet. This study aims to evaluate the *LBX1* methylation level in deep paravertebral muscles in order to analyze its association with IS occurrence and/or IS severity.

## 2. Materials and Methods

### 2.1. Patients

The study group consisted of 57 patients (50 girls, 7 boys) with IS. All patients underwent posterior spinal surgery correction for IS. The control group consisted of 20 patients (11 girls, 9 boys) with nonidiopathic spine deformities: spondylolisthesis, n = 2; congenital spine deformation, n = 14 (congenital scoliosis, congenital kyphosis); scoliosis secondary to prior thoracic surgery (lateral thoracotomy), n = 2; or Scheuermann’s disease, n = 2. All controls underwent posterior spinal surgery due to spine disorders (other than IS). All surgeries were performed in one hospital in a European country (Poland) from January 2017 until December 2019. All patients (both from the study group and control group) were subjected to a clinical, radiological, and molecular examination. The patients underwent standing anteroposterior radiographs before surgery. The curve pattern (number and localization of the curvatures), major curve angle (measured according to Cobb’s method) [34], and Risser sign (the radiological sign of skeletal maturity) [35] were measured in all patients by an experienced spine surgeon. The patients without coronal deformity (Scheuermann’s disease and spondylolisthesis) were not included in the major curve angle calculation.

The inclusion criteria for the study group were as follows: (1) clinically and radiologically confirmed IS diagnosis, (2) no coexisting orthopedic, genetic, or neurological disorders, (3) primary thoracic spinal curvature, and (4) surgical treatment due to IS. The study group was divided into two subgroups according to disease severity. The first subgroup consisted of 28 patients with a moderate form of IS, with curvatures ranging from 50° to 70° and a Risser sign of ≥4 and age of ≥15 years old. The second subgroup consisted of 29 patients with a very progressive form of IS, with larger curvatures exceeding 70° regardless of Risser’s sign of age.

The inclusion criteria for the control group were as follows: (1) clinically and radiologically confirmed diagnosis of congenital spine deformation with (defects of formation or segmentation), spondylolisthesis, scoliosis secondary to prior thoracic surgery, or Scheuermann’s disease, (2) no coexisting orthopedic, genetic, or neurological disorders, (3) no diagnosis of IS, and (4) surgical treatment due to spine pathology. 

### 2.2. Tissue Samples

During the surgery, three muscle tissue samples (1 cm^3^ each) were obtained from each patient via the surgical approach used to correct the deformity. The first sample was obtained from the deep paravertebral muscles (*M. longissimus*) on the (1) convex side at the apex of the major curvature. The second sample was obtained from the deep paravertebral muscles (*M. longissimus*) on the (2) concave side at the apex of the major curvature. The third sample was obtained from the (3) superficial muscle layer (*M. deltoideus*).

During the surgery, three muscle tissue samples (1 cm^3^ each) were obtained from each patient from the control group. Two were taken from the deep paravertebral muscles (*M. longissimus*). When curvature was present (congenital scoliosis), the samples were taken as follows: (1) on the convex and (2) concave sides at the apex of the major curvature. For patients with kyphosis or spondylolisthesis, the samples were taken as follows: (1) on the right side of the spine and (2) on the left side of the spine. The third sample was obtained in all patients from the (3) superficial muscle layer (*M. deltoideus*).

All samples were stored in sterile tubes containing 5 mL nucleic acid stabilizing solution (Novazym, cat no. ST01; Poznan, Poland).

### 2.3. Genomic DNA Methylation Analysis

Total genomic DNA was extracted and processed as described before [36]. The DNA was bisulfite converted and used as a template for polymerase chain reaction (PCR) followed by pyrosequencing (PSQ). The DNA sequences analyzed corresponded to the forward and reverse DNA strands of the *LBX1* promoter region (https://www.ncbi.nlm.nih.gov (accessed on 31 March 2018); GenBank N°: NG_009236). The PCR primers used are shown in Table 1. 

PCR reactions were performed using conditions validated for ZymoTaq^TM^ PreMix (Zymo Research; cat no. E2004; Irvine, CA, USA) [36]. Reaction mixture components, concentrations, and thermal profiles are presented in Table 2.

PSQ analysis was performed using the PyroMark Q48 instrument (Qiagen; Hilden, Germany) for complementary CpG dinucleotides (located in opposite strands of DNA). Assays were designed with Pyromark Q48 Autoprep 2.4.2 software (Qiagen; Hilden, Germany). For each strand, 23 CpG sites were analyzed. In each reaction, internal sodium bisulfite treatment quality control was included. The methylation level was quantified using Pyromark Q48 Autoprep 2.4.2 software and expressed as a percentage ratio of methylated to nonmethylated dinucleotides.

### 2.4. Statistical Analysis

Data analyses were performed using Statistica 13.3 software (TIBCO Software Inc.; Palo Alto, CA, USA) and PQStat 1.8.0.414 software (PQStat software; Poznan, Poland). The methylation level was analyzed in both strands separately for each CpG site and compared between selected subgroups. The Shapiro–Wilk test was used for the normality of continuous variable distribution assessment. The differences in methylation levels between concave, convex, and superficial muscles were evaluated using Friedman ANOVA with Dunn’s Bonferroni post hoc test. Methylation between patient subgroups with a major curve angle of ≤70° or >70° was compared using the Mann–Whitney U test. Data were considered statistically significant when *p* < 0.05. 

## 3. Results

### 3.1. Patients and Controls

The patient group consisted of 57 IS patients (7 boys and 50 girls), and the control group consisted of 20 individuals (9 boys and 11 girls). The mean age at surgery for patients was 14.1 ± 1.6 years (ranging from 11 to 18 years), and for controls was 13.6 ± 3.2 years (ranging from 7 to 18 years), and there were no significant differences between groups (*p* > 0.05). The major curve angle for patients ranged from 50° to 115°, with a mean of 76 ± 17°, and for controls, the major curve angle value ranged from 30° to 105°, with a mean of 64 ± 24°.

### 3.2. DNA Methylation at the LBX1 Promoter Regions—A Case-Control Study

**Figure 1:**
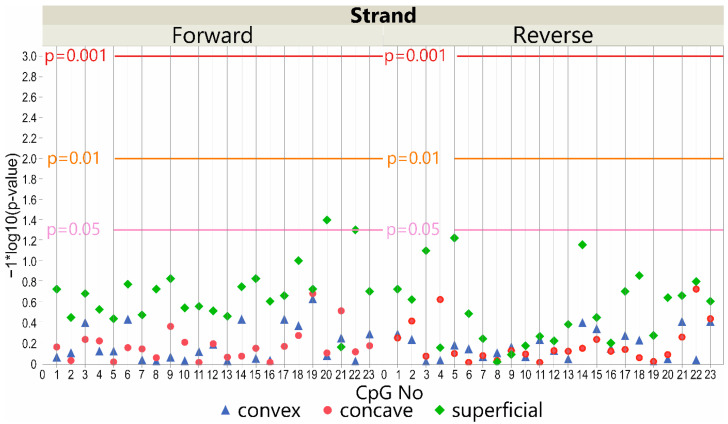
Scatter plot of −log10 *p*-values showing the difference between controls and patients in methylation level at *LBX1* DNA forward strand (left) and DNA reverse strand (right) promoter CpG sites in deep convex (blue triangles), deep concave (red triangles), and superficial (green diamonds) muscles. Reference horizontal lines represent the *p*-values.

The methylation level within the *LBX1* DNA forward strand promoter region differed slightly between patients and controls for the superficial muscles used as our internal control but not for the deep muscles. In particular, a significantly lower methylation level was observed in the superficial muscles of the control group at one site, CpG-20 (*p* < 0.05, Figure 1; Additional file 1: Table S1; Additional file 2: Figure S1). There were no significant differences observed in the DNA reverse strand promoter region (*p* > 0.05, Figure 1; Additional file 1: Table S2; Additional file 2: Figure S2).

### 3.3. DNA Methylation at the LBX1 Promoter Regions—Deep Paravertebral Muscles vs. Superficial Muscles

#### 3.3.1. DNA Forward Strand Promoter Region

**Figure 2:**
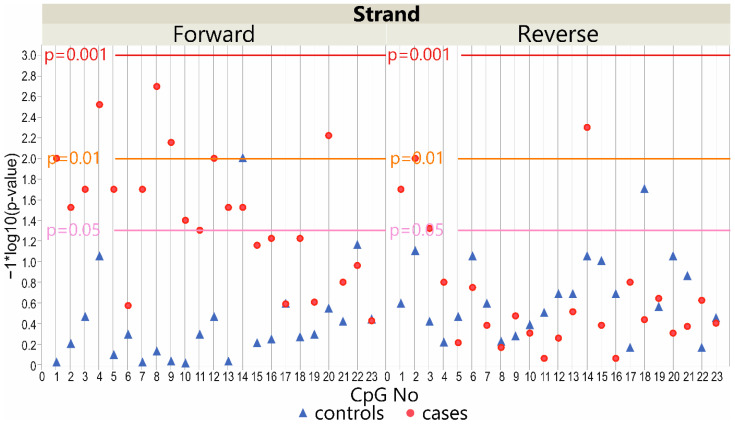
Scatter plot of −log10 *p*-values showing the difference between methylation level at *LBX1* DNA forward strand (left) and DNA reverse strand (right) promoter CpG sites in cases and controls (convex vs. concave vs. superficial muscles). Reference horizontal lines represent the *p*-values.

Considering the IS patients’ group, the methylation level within the *LBX1* DNA forward strand promoter region differed significantly between the deep paravertebral muscles (on the convex and concave side of the curvature) and the superficial muscles (*p* < 0.05; Figure 2). The methylation level was significantly higher in the superficial muscle compared with the convex side of the curvature in 13 CpG sequences (*p* < 0.05; Additional file 1: Table S1; Additional file 2: Figure S1). A significantly higher methylation level was also observed in the superficial muscle compared to the concave side of the curvature at CpG-3 and CpG-14 sites (*p* < 0.05; Additional file 1: Table S1; Additional file 2: Figure S1). However, there was no difference in the methylation level of the DNA from the deep paravertebral muscles between the convex or concave side of the curvature (*p* > 0.05; Additional file 1: Table S1; Additional file 2: Figure S1).

In the control group, a significant difference in methylation level was observed between the superficial muscles compared with the convex side deep muscles of the curvature at CpG-14 (*p* < 0.05, Figure 2; Additional file 1: Table S1; Additional file 2: Figure S1). In the deep paravertebral muscles, the methylation level differed significantly between the convex and concave sides of the curvature at CpG-14 (*p* < 0.05, Figure 2; Additional file 1: Table S1; Additional file 2: Figure S1). 

#### 3.3.2. DNA Reverse Strand Promoter Region

Considering the patients’ group, the methylation level within the *LBX1* DNA reverse strand promoter region was significantly different between deep paravertebral muscles (on the convex and concave sides of the curvature) and superficial muscles (*p* < 0.05, Figure 2). The methylation level was higher in the superficial muscle tissue compared with the deep convex muscles of the curvature in four CpG sequences (*p* < 0.05; Additional file 1: Table S2; Additional file 2: Figure S2). In the control group, no difference was observed (*p* > 0.05; Additional file 1: Table S2; Additional file 2: Figure S2). The methylation level differed significantly at CpG-18 between the superficial muscles and the concave side of scoliosis relative to the control group (*p* > 0.05; Additional file 1: Table S2; Additional file 2: Figure S2). A significantly higher methylation level was observed in the convex side of the curvature compared with the concave one at CpG-1 and CpG-2 within the patients’ group (*p* < 0.05; Additional file 1: Table S2; Additional file 2: Figure S2). In the control subgroup, the methylation level did not differ significantly between these muscles (Additional file 1: Table S2; Additional file 2: Figure S2).

### 3.4. LBX1 Methylation Status and Major Curve Angle—Case-Only Study

#### 3.4.1. DNA Forward Strand Promoter Region

**Figure 3:**
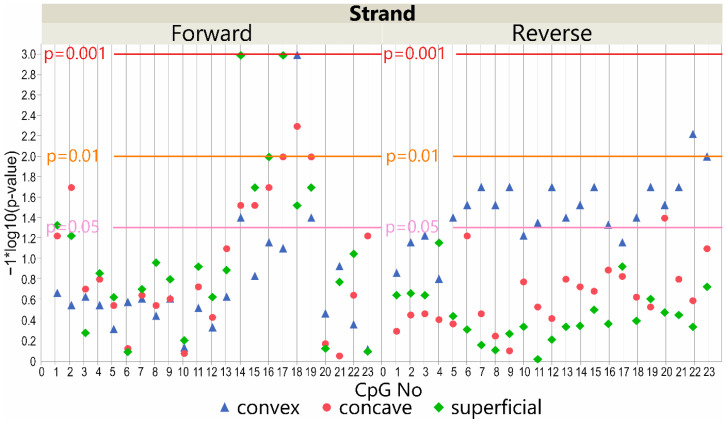
Scatter plot of −log10 *p*-values showing the difference between patients with major curve angle ≤70° and >70° in methylation level at *LBX1* DNA forward strand (left) and DNA reverse strand (right) promoter CpG sites in deep convex (blue triangles), deep concave (red triangles), and superficial (green diamonds) muscles. Reference horizontal lines represent the *p*-values.

In the study group, we observed significant differences between case subgroups with a major curve angle of ≤70° vs. >70°. The methylation level was higher in patients with a major curve angle of >70° (*p* < 0.05; Additional file 1: Table S3; Additional file 2: Figure S3). The methylation from DNA isolated at the convex side of the curvature differed at three CpG sites on the forward strand (*p* < 0.05, Figure 3). In seven CpG sequences, differences were also detected on the forward strand of the concavity of thoracic scoliosis and the superficial muscles (*p* < 0.05, Figure 3; Additional file 1: Table S3; Additional file 2: Figure S3). 

#### 3.4.2. DNA Reverse Strand Promoter Region

For the study group, we mainly observed significant differences between cases with a major curve angle of ≤70° vs. >70° on the convex side of the curvature. The methylation level was higher in patients with a major curve angle of >70° in 17 of 23 analyzed CpG sequences (*p* < 0.05, Figure 3; Additional file 1: Table S4; Additional file 2: Figure S4). The methylation at the concave side of the curvature differed only in one CpG and was lower in patients with a major curve angle of ≤70° (*p* < 0.05, Figure 3; Additional file 1: Table S4; Additional file 2: Figure S4). No differences were observed in the superficial muscles (*p* > 0.05, Figure 3; Additional file 1: Table S4; Additional file 2: Figure S4). 

### 3.5. LBX1 Methylation Status and Major Curve Angle—Deep Paravertebral Muscles vs. Superficial Muscles

#### 3.5.1. DNA Forward Strand Promoter Region

**Figure 4:**
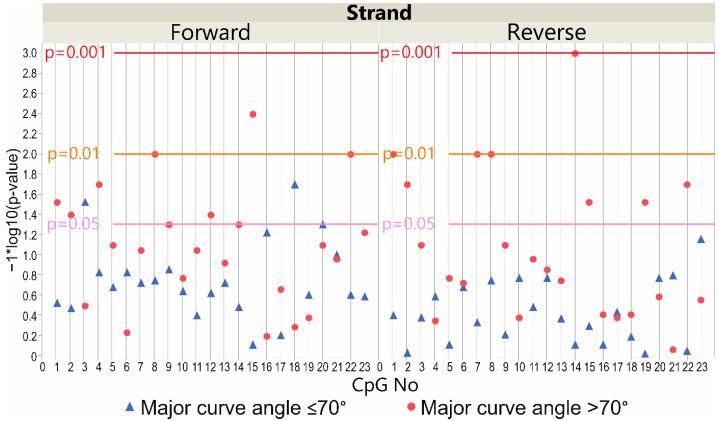
Scatter plot of −log10 *p*-values showing the difference between methylation level at *LBX1* DNA forward strand and DNA reverse strand promoter CpG sites in patients with major curve angle ≤70° and >70° (convex vs. concave vs. superficial muscles). Reference horizontal lines represent the *p*-values.

Considering the patients’ subgroup with a major curve angle of ≤70°, the methylation level within the *LBX1* DNA forward strand promoter region differed significantly between deep paravertebral muscles (on the convex and concave sides of the curvature) and superficial muscles in two CpG sites (*p* < 0.05, Figure 4). The methylation level was significantly higher in the superficial muscle compared with the concave side of the curvature at CpG-3 and CpG-18 (*p* < 0.05; Additional file 1: Table S3; Additional file 2: Figure S3). A significantly higher methylation level was also observed in the superficial muscle compared with the convex side of the curvature at the same CpG sites (*p* < 0.05; Additional file 1: Table S3; Additional file 2: Figure S3). 

There was a difference in the patients’ subgroup with a major curve angle of >70° regarding the methylation level in deep paravertebral muscles on the convex and concave sides of the curvature and the superficial muscles in seven CpG sequences (*p* < 0.05, Figure 4). A higher methylation level was observed in the superficial muscle compared with the convex side of the curvature at CpG sites: 1, 2, 4, 8, 12, 15, and 22 (*p* < 0.05; Additional file 1: Table S3; Additional file 2: Figure S3).

#### 3.5.2. DNA Reverse Strand Promoter Region

Considering the patients’ subgroup with a major curve angle of ≤70°, we observed no differences in the methylation level within the *LBX1* DNA reverse strand promoter region (*p* > 0.05, Figure 4). 

There was, however, a difference in the patients’ subgroup with a major curve angle of >70° in regard to the methylation level in deep paravertebral muscles on the convex and concave sides of the curvature and the superficial muscles in eight CpG sequences (*p* < 0.05, Figure 4). A higher level of methylation was observed in the convexity of scoliosis compared with the concave side of the curvature at CpG sites: 1, 2, 7, and 8 (*p* < 0.05; Additional file 1: Table S4; Additional file 2: Figure S4). A higher level of methylation was also observed in the convex side of the curvature compared with the superficial muscle at CpG sites: 1, 7, 8, 14, 15, 19, and 22 (*p* < 0.05; Additional file 1: Table S4; Additional file 2: Figure S4). A higher level of methylation was observed in the concave side of the curvature compared to the superficial muscle at CpG-14 (*p* < 0.05; Additional file 1: Table S4; Additional file 2: Figure S4).

## 4. Discussion

Although many studies concerning the etiology of IS have been conducted, the IS background remains unsolved [4,37,38]. Multiple theories concerning IS etiology have been suggested. These concepts cover, e.g., aberrations in hormonal level, metabolic factors, connective tissue, skeletal and muscle structure, biomechanical features, neurological mechanisms, molecular and genetic factors, biochemistry, environment, lifestyle, or possible interrelationships among them [4,37,38,39].

In this study, we focused on muscle tissue as a scoliosis-inducing factor. However, we cannot exclude another important background, such as a relative anterior spinal overgrowth. It may induce rotational instability and functional tethering of the spinal cord [40,41,42].

This study revealed a new factor associated with a tendency to scoliosis progression. We found an association between *LBX1* methylation level and disease severity. Understanding the background of the disease can contribute to the prediction of the IS course in patients and may create the possibility of prevention or moderate treatment.

Although we assumed that *LBX1* promoter methylation might be associated with a predisposition to IS, our results did not confirm this concept. No difference in the methylation level between IS patients versus controls at evaluated CpG sequences in deep paravertebral muscles was found. What is more, we found one significant difference in CpG methylation on the forward strand in superficial muscles (Figure 1). We included the evaluation of the superficial muscles in our study as an internal control. We believe that the superficial muscles are not associated with IS onset due to their function and the anatomic borderlines between them and the deep paravertebral muscles. Thus, we do not consider them an important causative factor.

The most important finding of our study is a higher methylation level in the *LBX1* promoter region on the reverse strand at the deep convex muscles in patients with more severe IS. We found significant differences in 17 out of 23 analyzed CpG sequences and a tendency in almost all CpG sequences (Figure 3). The methylation was also higher in the subgroup of patients with greater curvatures on the forward strand. However, this difference was present in all muscle layers in several CpG sequences and seemed to be more diffused. Interestingly, results for CpG-14, CpG-18, and CpG-19 were analogous, regardless of the tissue location (Figure 3).

The analysis of disease severity is essential for IS treatment. It is unknown why certain IS patients progress more than others. Therefore, identifying the patients at risk of scoliosis occurrence or curve progression is crucial to providing early treatment [1,43].

To evaluate the association of the *LBX1* gene methylation level with IS progression, we divided the study group into two subgroups according to disease severity. Unfortunately, there is no defined major curve angle threshold when severe curvature significantly impacts patients’ health. It is established that severe curvatures affect patients’ health, such as decreased lung function, cardiac function, back pains, and degenerative spine disease [44,45,46]. A correlation between the degree of patients’ impairment with the severity of the spinal deformity was revealed [3,44,45]. The group of skeletally mature patients with a major curve angle moderately exceeding 50° needs a surgical scoliosis correction to avoid further curvature deterioration in adulthood, while in patients with a bigger curve angle, direct impairment can be found [2,46,47]. Studies concerning surgical scoliosis treatment classify severe curvature as a major curve angle exceeding 70° [48,49,50]. Thus, we used this value to categorize study subgroups. The inclusion criteria of a Risser sign of ≥4 and age of ≥15 years old were used for the subgroup of patients with a moderate form of IS—having the curvature range from 50° to 70°. The rationale was to ensure that these patients developed the final major curve angle during the natural history of the curvature. 

A difference in the *LBX1* methylation level between the muscle layers is another interesting finding in the context of IS background. Within the patients’ group, we found a tendency for higher DNA methylation levels in superficial muscles compared with deep muscles. This difference was significant in 13 and 4 CpG sequences on the forward and reverse DNA strands, respectively. This issue was less pronounced in the control group and was present in one CpG on both forward and reverse strands. Thus, we found a different *LBX1* methylation level between the muscle tissue harvested from different localizations (*M. longissimus* vs. *M. deltoideus*). It raises doubt about the possibility of using *LBX1* methylation for prediction in IS patients based on samples from different tissues, such as blood. It is difficult to distinguish the causative factor from the effect of the disease. The difference in methylation could be the cause of the asymmetry in muscles, which may contribute to the etiology of IS. Conversely, differences in methylation levels could be a consequence of the muscles being exposed to different conditions on either side of the curvature due to asymmetric loading. The difference in methylation level between the groups is found mostly in deep paravertebral muscles on the convex side at the reverse DNA strand *LBX1* gene promoter region. The difference is less demonstrated on the forward strand and is more equally spread in all muscles. In case of secondary changes, we could expect more similar distribution of methylation at both DNA strands. Thus, we can assume that it is rather a causative factor.

A theory of IS background connecting the impact of dysfunctional paravertebral muscles on the development of spine curvature has been proposed [51,52]. Differences in the proportion of muscle fiber types between convex and concave sides of IS were described: a lower percentage of slow fibers type I in IS patients than in controls was found [53]. Other studies described greater fibrosis and fatty involution on the deep concave paravertebral muscles than on the convex side [51,54]. What is more, asymmetry of muscle activation patterns in IS patients between the concave and convex sides has also been described [55,56]. We found differences in the *LBX1* gene DNA methylation level in deep paravertebral muscles between the analyzed groups. DNA methylation does not directly affect muscles but impacts *LBX1* gene expression. LBX1 acts primarily during embryogenesis, and its asymmetric expression in relation to biological action is difficult to establish [12,13,14]. We do not know if the difference between both sides of the curvature could modulate muscle strength or elasticity. Nevertheless, it might be of important clinical relevance; unfortunately, this study did not evaluate muscle tissue properties.

To our knowledge, one study concerning *LBX1* gene expression in paravertebral muscles has been published [13]. Jennings et al. evaluated deep paravertebral muscles from 25 IS patients. The patients evaluated by Jennings et al. were comparable to ours by age and gender, while our group presented a slightly higher major curve angle value. They found no difference in mRNA and protein expression between the concave and convex sides of the curvature. What is more, the results did not correlate with the major curve angle. However, no subgroup analysis according to curve severity was conducted. Because *LBX1* expression was not evaluated in this study, direct comparison is difficult. When all patients were pooled together, the paired analysis did not find a convex–concave difference in the *LBX1* methylation level on the forward strand. A difference was only observed in two CpG sequences on the reverse strand. It is comparable with Jennings et al.’s description of the expression pattern. However, we found a significant difference associated with curve severity. Jennings et al. theorized that *LBX1* expression occurs in the time of ontogeny and IS-associated *LBX1* genetic variants modify gene expression unequally on both sides of the scoliotic curve during the embryogenesis of muscle cells. Still, this expression aligns after birth [13]. It could result from the different methylation patterns on the forward and reverse DNA strands revealed in our study. It is possible that at distinct stages of development, the forward or reverse strand is more relevant to gene expression.

Previous work has shown an association between DNA methylation and IS. However, all these studies used peripheral blood samples. Liu and colleagues, using whole-genome methylation evaluation in a twin pair, selected DNA regions potentially associated with adolescent IS and described a significantly higher methylated region in chromosome 15 [30]. Meng et al. described an association between lower methylation levels at site cg01374129 and curve severity [26]. According to Shi et al., susceptibility to IS and severity of the curvature are associated with the level of methylation of the *PITX1* and *PCDH10* genes [32,33]. Mao et al. evaluated the methylation level of the *COMP* gene. They found that hypermethylation of the gene promoter correlated with curve severity [31].

Taking into consideration that our study was focused on a local evaluation in search of a causative factor in muscle tissue, it is difficult to compare it with the results obtained using blood samples, especially as we found a difference in methylation levels between the deep and superficial groups of muscles in IS patients. Thus, the level of *LBX1* methylation in blood samples may be vastly different from that revealed in muscles.

One of the biggest challenges of this study was to find a suitable control group. This study was focused on paraspinal muscle tissues, and it was unreasonable, because of ethical issues, to obtain paraspinal muscle samples from healthy individuals. We decided not to include elderly patients who underwent surgery due to degenerative spine changes, which may cause muscle atrophy or unknown methylation changes. What is more, there would be an important difference in age. Thus, we decided to include into the control group patients of similar age, including children operated on due to spine abnormalities for known reasons and excluding patients with scoliosis due to a neuromuscular or genetic background. Spinal pathologies requiring surgical treatment in children meeting the inclusion criteria are much rarer than IS. Thus, the control group is smaller than the IS patients’ group. Patients with congenital scoliosis and scoliosis after surgical thoracic surgery had a mean Cobb 12° smaller than IS patients. Two patients with Scheuermann’s disease and two patients with spondylolisthesis were not included in the major curve angle calculation due to deformation in the sagittal plane, and they did not present scoliosis. The most significant differences between the groups were found in the gender distribution. However, it is a known phenomenon that severe IS is much more common in females, and the prevalence of congenital scoliosis did not differ between boys and girls [57]. What is more, we did not find an association between *LBX1* and gender. Thus, we decided to accept this difference.

Patients with congenital scoliosis were the largest part of the control group. While evaluating our results, it is important to notice that there are studies describing genetic predisposition in congenital scoliosis [58,59,60]. Several studies have reported several genes associated with congenital scoliosis, including *TBX6* [58,59], *FBN1* [61], *PAX1* [62], *DLL1* [63], and other genes. However, to our knowledge, the association between congenital scoliosis and the *LBX1* gene has never been found. However, to our knowledge, the association between congenital scoliosis and the *LBX1* gene has never been found. What is more, in genetic studies evaluating tissue samples of patients with idiopathic scoliosis, the patients with congenital scoliosis have been used as a control group [64,65,66].

The strength of this study is that it links known facts about the genetic background of IS concerning *LBX1* with possible modification of its expression or function in the area of deformation. This brings a new insight into IS pathology and evaluates a probable cause of curvature progression.

## 5. Conclusions

The association between *LBX1* promoter methylation and IS severity was demonstrated. In patients with severe IS, the deep paravertebral muscles demonstrate an asymmetric *LBX1* promoter region methylation level, higher at the convex scoliosis side, which reveals the role of locally acting factors in IS progression.

## Figures and Tables

**Table 1:** Primer sequences designed using PyroMark Assay Design software (version 2.0.1.15; Qiagen; Hilden, Germany).

	Primer	Sequence	Length (nt)	Tm (°C)	GC (%)	PCR Product Size
*LBX1* Forward	^B^→ PCR	TTTAGGTAGTGGGGTGAG	18	55.8	50.0	256 bp
	← PCR	CCCCAACTATTTATAAATTACATTAACTAC	30	51.9	26.7	
	← SEQ	ATAAATTACATTAACTACTCCTT	23	44.0	21.7	-
*LBX1* Reverse	→ PCR	GTAGTGGGGTGAGGGGTAA	19	60.3	57.9	333 bp
	← ^B^ PCR	ACATTAACTACTCCTTTATTACACC	25	57.2	32.0	
	→ SEQ	GAGGGGTAAGAGGGT	15	50.8	60.0	-

→ PCR, forward primer; ← PCR, reverse primer; ^B^, biotinylated primer; Tm, melting temperature; GC, guanine–cytosine content; bp, base pairs; → SEQ, forward sequencing primer; ← SEQ, reverse sequencing primer.

**Table 2:** PCR mixture content and thermal profile of the reactions (data adapted from previously published paper [36]).

Component	Initial Concentration	Volume Added	Final Concentration	Mixture Volume
ZymoTaq^TM^ Premix	2×	5 µL	1×	10 µL
→ PCR	10 µM	1 µL	1 µM	
← PCR	10 µM	1 µL	1 µM	
DNA	100 ng/µL	0.2 µL	2 ng/µL	
Nuclease-free water		2.8 µL		
Thermal profile of the reactions				
Number of cycles	Step		Duration, temperature	
1	Initial denaturation		10 min, 95 °C	
37	Denaturation		30 s, 95 °C	
	Annealing		30 s, 54 °C ^A^, 58 °C ^B^	
	Extension		60 s, 72 °C	
1	Final extension		7 min, 72 °C	
1	Hold		∞, 4 °C	

→ PCR, forward primer; ← PCR, reverse primer; min, minutes; s, seconds; ^A^, for forward strand; ^B^, for reverse strand.

## Data Availability

The datasets used and analyzed during the current study are available from the corresponding author on reasonable request.

## Supplementary Materials

The following supporting information can be downloaded at: https://www.mdpi.com/article/10.3390/genes13091556/s1, Additional file 1: Table S1: methylation level at *LBX1* DNA forward strand promoter region in control and patient subgroups in deep paravertebral muscles and superficial muscles, Table S2: methylation level at *LBX1* DNA reverse strand promoter region in control and patient subgroups in deep paravertebral muscles and superficial muscles, Table S3: methylation level at *LBX1* DNA forward strand promoter region in patient subgroups with major curve angle ≤70° and >70° in deep paravertebral muscles and superficial muscles, Table S4: methylation level at *LBX1* DNA reverse strand promoter region in patients subgroup with major curve angle ≤70° and >70° in deep paravertebral muscles and superficial muscles, Additional file 2: Figure S1: DNA methylation level within *LBX1* forward strand promoter region in deep paravertebral muscles and superficial muscles. Upper solid horizontal lines represent significant differences between muscles, and lower lines show significant differences in case-control study, Figure S2: DNA methylation level within *LBX1* reverse strand promoter region in deep paravertebral muscles and superficial muscles. Upper solid horizontal lines represent significant differences between muscles, and lower lines show significant differences in case-control study, Figure S3: DNA methylation level within *LBX1* forward strand promoter region in deep paravertebral muscles and superficial muscles. Upper solid horizontal lines represent significant differences between muscles in case of patients, and lower lines show significant differences in subgroups divided according to major curve angle values, Figure S4: DNA methylation level within *LBX1* forward strand promoter region in deep paravertebral muscles and superficial muscles. Upper solid horizontal lines represent significant differences between muscles in case of patients, and lower lines show significant differences in subgroups divided according to major curve angle values.
