# Supplementary material for: Association of LBX1 Gene Methylation Level with Disease Severity in Patients with Idiopathic Scoliosis: Study on Deep Paravertebral Muscles
Source: Genes (Basel). 2022 Aug 29;13(9):1556. doi: 10.3390/genes13091556 (PMC9498322; doi:10.3390/genes13091556)
Supplement: Supplementary file 1 [file genes-13-01556-s001.zip › genes-1837166-supplementary/Additional file 1.pdf]

Table S1. Methylation level at *LBX1* DNA forward strand promoter region in control and patients subgroups in deep paravertebral muscles and superficial muscles.

CpG	Group	N	Convex									Concave									Superficial									*p*-value[B]	Post-hoc
			M	Me	Min	Max	Q1	Q3	SD	SE	p-value[A]	M	Me	Min	Max	Q1	Q3	SD	SE	p-value[A]	M	Me	Min	Max	Q1	Q3	SD	SE	*p*-value[A]		
1	controls	20	13.17	13.21	7.06	26.85	9.94	15.17	4.66	1.04	0.89[a]	13.01	12.83	6.29	22.62	10.54	15.50	3.72	0.83	0.70[a]	12.95	12.93	5.41	22.10	9.52	14.55	4.42	0.99	0.19[a]	0.97[c]	
	cases	57	13.02	12.94	5.61	21.64	10.33	16.22	3.84	0.51		13.41	13.16	6.46	24.08	11.01	15.46	3.98	0.53		14.44	13.69	6.37	25.21	11.91	17.70	4.32	0.57		**0.01**[c]	**0.01**[¥,f]
2	controls	20	13.33	13.42	6.63	27.03	10.74	14.63	4.36	0.98	0.81[b]	13.26	13.91	7.12	21.70	11.04	15.05	3.48	0.78	0.95[a]	13.04	12.53	5.28	23.68	9.79	15.87	4.58	1.02	0.36[a]	0.64[d]	
	cases	57	12.83	12.79	4.13	20.24	10.07	16.26	3.99	0.53		13.32	13.37	5.84	22.59	11.10	15.30	3.73	0.49		14.10	13.21	5.87	25.51	11.18	17.39	4.32	0.57		**0.03**[c]	**0.02**[¥,f]
3	controls	20	15.11	14.53	9.01	29.09	13.06	16.81	4.37	0.98	0.41[b]	15.88	15.63	10.20	27.06	13.59	17.79	4.15	0.93	0.59[a]	15.22	15.32	7.07	25.23	12.57	17.63	4.39	0.98	0.21[a]	0.35[d]	
	cases	57	15.38	15.13	6.21	26.85	12.54	17.85	4.25	0.56		15.33	15.36	7.79	22.29	12.96	18.26	3.74	0.50		16.57	16.50	8.30	27.73	14.37	18.61	3.98	0.53		**0.02**[c]	**0.04**[¥,f]**; 0.03**[£,f]
4	controls	20	17.75	17.29	9.87	35.13	14.70	19.51	5.60	1.25	0.78[b]	18.87	18.22	10.52	33.80	16.62	21.95	5.15	1.15	0.61[a]	17.92	17.21	9.56	29.98	13.72	22.18	5.85	1.31	0.30[a]	0.09[d]	
	cases	57	17.71	17.91	7.43	27.79	13.56	21.39	4.87	0.65		18.23	18.45	7.76	26.89	15.25	21.44	4.72	0.62		19.39	19.02	7.44	30.61	16.38	22.99	5.20	0.69		**0.003**[c]	**0.003**[¥,f]
5	controls	20	16.65	15.54	8.27	30.77	14.57	17.81	4.96	1.11	0.78[b]	16.88	16.44	10.62	29.64	14.61	18.48	4.16	0.93	0.98[b]	16.75	15.36	9.09	27.67	14.47	19.42	4.92	1.10	0.37[a]	0.82[d]	
	cases	57	16.51	16.62	6.23	26.89	14.03	19.74	4.33	0.57		16.65	16.48	7.68	25.25	14.21	18.80	4.22	0.56		17.86	17.70	7.84	31.97	14.50	21.03	4.69	0.62		**0.02**[c]	**0.03**[¥,f]
6	controls	20	16.57	15.78	8.00	32.80	12.41	19.34	6.08	1.36	0.38[b]	17.30	17.33	8.91	33.01	13.00	18.70	5.70	1.27	0.71[b]	16.75	14.72	8.22	25.69	12.15	22.76	5.87	1.31	0.17[a]	0.52[d]	
	cases	57	17.50	17.33	5.34	36.22	13.58	20.67	6.17	0.82		18.24	16.98	6.76	42.66	13.44	23.06	6.75	0.89		18.76	18.95	9.11	32.43	14.51	21.98	5.60	0.74		0.27[d]	
7	controls	20	16.93	17.85	6.82	36.21	12.17	20.01	6.41	1.43	0.95[a]	17.04	16.24	8.42	30.34	13.78	20.34	5.10	1.14	0.73[a]	17.08	16.77	8.26	28.24	12.81	20.65	5.77	1.29	0.34[a]	0.97[e]	
	cases	57	16.84	15.73	4.84	28.30	13.47	20.53	5.16	0.68		17.53	18.42	6.25	28.69	12.69	20.42	5.55	0.74		18.44	18.28	7.76	29.17	14.46	22.52	5.28	0.70		**0.02**[e]	**0.01**[¥,f]
8	controls	20	23.64	22.33	10.66	47.59	16.87	28.45	8.54	1.91	0.98[a]	24.39	24.57	13.15	43.82	19.02	28.60	7.30	1.63	0.89[a]	23.54	22.59	11.69	39.83	16.55	30.04	8.67	1.94	0.19[a]	0.76[e]	
	cases	57	23.69	23.12	7.80	38.27	17.82	30.02	7.50	0.99		24.68	25.24	8.00	42.34	18.63	28.07	7.96	1.05		26.33	26.06	11.57	42.44	19.95	33.16	7.99	1.06		**0.002**[c]	**0.001**[¥,f]
9	controls	20	17.76	17.45	8.77	35.76	14.44	20.88	5.83	1.30	0.89[b]	17.54	17.11	9.77	33.94	13.50	20.30	5.22	1.17	0.44[b]	17.28	16.57	9.52	30.17	12.59	21.40	5.62	1.26	0.15[a]	0.95[d]	
	cases	57	17.60	17.58	6.21	29.09	13.66	22.19	4.96	0.66		18.19	18.15	8.40	28.05	14.39	22.24	5.01	0.66		19.24	18.68	9.67	29.70	15.33	22.88	5.02	0.66		**0.007**[c]	**0.005**[¥,f]
10	controls	20	24.30	23.50	12.44	44.66	19.98	26.94	7.05	1.58	0.97[a]	24.46	24.20	15.05	40.19	20.40	27.31	5.95	1.33	0.63[a]	24.29	23.69	13.32	40.58	18.64	28.89	6.88	1.54	0.29[a]	0.99[c]	
	cases	57	24.23	24.58	8.36	37.01	19.25	28.84	6.74	0.89		25.28	25.20	9.86	43.54	20.62	31.28	6.83	0.90		26.07	25.03	14.46	38.61	21.10	31.07	6.32	0.84		**0.04**[c]	**0.03**[¥,f]
11	controls	20	21.59	21.25	14.66	38.01	18.16	23.35	5.22	1.17	0.79[b]	22.11	21.72	16.49	34.99	19.33	24.03	4.18	0.94	0.99[b]	21.41	19.96	12.54	33.19	17.12	25.95	5.56	1.24	0.28[a]	0.52[c]	
	cases	57	21.55	21.75	9.40	32.92	18.06	25.23	5.07	0.67		22.18	21.86	9.79	35.83	17.21	25.95	5.62	0.74		22.85	21.87	14.73	32.88	18.91	25.83	4.94	0.65		0.05[e]	
12	controls	20	21.23	21.10	12.86	38.68	17.67	23.26	5.49	1.23	0.67[b]	22.20	21.97	15.16	36.84	19.05	24.27	4.75	1.06	0.65[b]	21.67	21.71	14.89	33.71	17.22	25.33	5.28	1.18	0.31[a]	0.35[c]	
	cases	57	21.42	21.23	10.10	31.11	17.59	25.09	5.24	0.69		22.30	22.36	11.92	33.43	17.76	26.36	5.39	0.71		23.01	23.06	13.49	32.98	19.21	26.07	4.94	0.65		**0.01**[e]	**0.008**[¥,f]
13	controls	20	23.40	23.59	14.87	38.07	19.82	25.96	5.29	1.18	0.97[a]	23.68	23.40	16.31	35.74	20.26	26.90	4.81	1.08	0.88[a]	23.43	23.01	15.77	33.53	19.04	27.29	5.18	1.16	0.35[a]	0.95[c]	
	cases	57	23.34	22.63	12.45	33.45	19.60	27.79	5.27	0.70		23.88	23.94	14.07	35.28	19.39	27.48	5.26	0.70		24.73	24.47	15.17	34.81	20.97	28.49	5.38	0.71		**0.03**[c]	**0.02**[¥,f]
14	controls	20	7.86	8.00	4.42	13.04	6.03	9.39	2.17	0.49	0.38[b]	8.57	8.64	4.85	13.22	6.56	9.95	2.49	0.56	0.86[b]	8.54	6.95	5.08	15.41	6.32	10.89	3.03	0.68	0.18[b]	**0.01**[d]	**0.02**[‡,g]**; 0.02**[¥,g]
	cases	57	8.69	8.04	4.20	16.44	6.68	10.13	2.80	0.37		8.83	8.54	4.88	16.56	6.32	10.56	2.86	0.38		9.46	8.45	4.59	16.86	7.24	11.20	3.27	0.43		**0.03**[d]	**0.046**[¥,h]**; 0.046**[£;h]
15	controls	20	29.39	29.46	18.63	49.58	21.89	35.03	8.24	1.84	0.92[b]	29.57	31.89	18.06	47.24	21.66	34.63	7.70	1.72	0.72[b]	28.66	27.78	16.02	47.65	21.01	35.60	9.36	2.09	0.15[b]	0.63[e]	
	cases	57	29.10	27.70	16.86	44.48	21.96	37.28	7.91	1.05		30.26	30.82	16.66	46.72	22.38	35.73	8.10	1.07		31.52	31.60	17.56	44.41	24.45	38.36	8.09	1.07		0.07[d]	
16	controls	20	24.95	24.79	16.48	43.49	20.37	28.82	6.32	1.41	0.96[b]	25.85	24.45	17.08	41.17	21.53	29.36	5.79	1.30	0.99[a]	24.88	23.17	16.35	39.82	19.61	29.40	6.93	1.55	0.25[b]	0.58[c]	
	cases	57	25.00	23.05	12.61	37.59	19.98	28.43	6.55	0.87		25.87	24.71	14.37	39.63	20.69	31.41	6.51	0.86		26.42	24.59	15.29	38.31	21.34	30.99	6.28	0.83		0.06[d]	
17	controls	20	11.96	13.22	4.44	20.71	9.26	14.49	4.10	0.92	0.38[b]	12.30	13.72	5.33	19.24	7.96	15.28	4.16	0.93	0.69[b]	11.46	13.22	2.82	18.96	6.92	14.90	4.82	1.08	0.22[b]	0.26[d]	
	cases	57	12.71	13.62	4.97	18.49	10.15	15.95	3.79	0.50		12.83	13.99	2.33	18.56	10.43	15.35	3.90	0.52		13.14	13.97	2.48	18.83	11.47	16.28	3.93	0.52		0.26[d]	
18	controls	20	8.94	8.15	4.58	15.76	6.41	11.86	3.18	0.71	0.44[b]	9.03	8.31	5.27	14.76	6.12	12.05	3.03	0.68	0.54[b]	8.60	7.76	4.76	15.56	6.12	10.83	3.19	0.71	0.10[b]	0.55[c]	
	cases	57	9.28	8.25	4.40	15.96	7.53	12.00	3.09	0.41		9.67	8.75	4.52	25.58	6.77	12.75	3.79	0.50		9.70	8.80	4.53	16.58	7.61	12.13	2.99	0.40		0.06[d]	
19	controls	20	9.05	7.88	5.48	15.31	6.46	11.74	3.07	0.69	0.24[b]	8.83	8.06	4.40	14.65	7.44	10.92	2.83	0.63	0.21[a]	8.95	7.68	4.05	15.03	6.91	12.21	3.23	0.72	0.19[b]	0.52[d]	
	cases	57	10.03	9.63	5.12	25.49	7.79	12.23	3.57	0.47		9.83	9.26	2.27	15.76	7.53	12.74	3.10	0.41		10.02	9.59	5.60	16.15	7.67	12.49	2.81	0.37		0.25[d]	
20	controls	20	17.81	17.28	11.83	29.46	15.33	19.51	3.78	0.84	0.86[b]	18.34	18.62	11.31	25.95	16.30	20.08	3.43	0.77	0.80[a]	16.74	16.73	10.51	25.85	14.23	19.71	3.95	0.88	**0.04**[a]	0.29[d]	
	cases	57	17.22	17.50	8.98	24.14	14.11	19.77	3.62	0.48		18.11	18.65	10.92	27.68	15.52	20.37	3.37	0.45		18.63	18.30	10.07	24.53	16.60	21.66	3.44	0.46		**0.006**[e]	**0.003**[¥,f]
21	controls	20	34.70	28.14	23.09	57.21	26.51	46.41	12.56	2.81	0.58[b]	36.53	29.82	22.27	62.13	27.33	51.24	13.04	2.91	0.31[b]	34.51	29.99	18.83	56.05	26.29	43.47	11.65	2.60	0.70[b]	0.39[d]	
	cases	57	32.33	28.54	19.49	59.24	25.13	32.72	11.28	1.49		32.73	29.31	21.32	59.15	25.41	32.98	11.20	1.48		33.25	29.71	21.04	68.82	26.78	33.61	11.57	1.53		0.16[d]	
22	controls	20	16.69	16.76	0.00	31.13	14.01	20.10	6.27	1.40	0.98[b]	18.30	17.18	0.00	30.66	16.23	21.81	6.18	1.38	0.78[b]	14.95	15.24	0.00	24.73	12.41	18.93	6.53	1.46	0.05[b]	0.07[d]	
	cases	57	17.04	16.97	9.59	25.70	13.70	20.34	4.13	0.55		17.79	17.32	9.16	25.42	15.59	20.19	3.87	0.51		18.16	18.25	0.00	32.34	15.25	21.25	5.11	0.68		0.11[c]	
23	controls	20	15.39	15.97	0.00	27.88	12.95	17.58	6.28	1.40	0.53[b]	16.91	16.87	0.00	27.37	13.39	21.32	6.53	1.46	0.68[b]	14.60	14.28	8.47	24.29	12.53	16.63	3.94	0.88	0.20[a]	0.37[c]	
	cases	57	16.82	16.18	0.00	57.38	11.53	19.52	7.87	1.04		17.17	16.65	0.00	74.72	13.07	20.37	9.29	1.23		16.26	15.84	0.00	24.63	12.42	21.08	5.24	0.69		0.38[d]	

Legend: [A] – Major curve ≤ 70° vs.>70° group p-value; [B] – concave vs. convex vs. superficial; [a] – t-Student test; [b] – Mann-Whitney U test; [c] - Repeated measures ANOVA test; [d] – Friedman rank test for repeated measures; [e] - Repeated measures ANOVA test with Greenhouse-Geisser correction; [f] – Tukey HSD post-hoc test [g] - Dunn Bonferroni-Hochberg post-hoc test; [h] – Benjamini-Hochberg post-hoc test; ; [i] – t-Student test with separate variance estimates; [j] – Fisher LSD post-hoc test [‡] - convex vs. concave; [¥] - convex vs. superficial; [£] - concave vs. superficial; Statistically significant differences are indicated in bold.

Table S2. Methylation level at *LBX1* DNA reverse strand promoter region in control and patients subgroups in deep paravertebral muscles and superficial muscles.

CpG	CpG	Group	N	Convex									Concave									Superficial									*p*-value^{B}	Post-hoc
				M	Me	Min	Max	Q1	Q3	SD	SE	p-value^{A}	M	Me	Min	Max	Q1	Q3	SD	SE	p-value^{A}	M	Me	Min	Max	Q1	Q3	SD	SE	p-value^{A}		
28	1	controls	20	32.63	31.90	11.42	54.12	26.41	36.77	11.26	2.52	0.53^{b}	33.98	33.99	14.11	53.21	28.57	40.26	10.67	2.39	0.57^{b}	35.51	33.50	12.80	56.51	26.19	43.65	12.17	2.72	0.19^{a}	0.26^{c}	
		cases	57	35.31	33.60	12.62	101.00	28.49	41.72	14.53	1.92		31.33	32.58	0.00	57.41	27.41	37.39	11.55	1.53		31.64	30.97	9.98	54.76	24.19	38.94	11.01	1.46		**0.02**^{d}	**0.049**^{‡,g}**;0.03**$^{¥,g}$
27	2	controls	20	24.97	23.02	12.73	41.10	19.95	28.41	8.27	1.85	0.60^{b}	22.07	21.74	6.53	35.57	17.99	27.61	7.60	1.70	0.39^{a}	24.93	23.93	14.14	34.67	19.61	31.18	6.29	1.41	0.24^{a}	0.08^{c}	
		cases	57	26.42	24.58	13.82	101.00	20.31	29.70	11.94	1.58		23.55	22.81	12.52	43.84	19.22	27.55	6.28	0.83		23.12	23.79	11.09	38.16	19.85	26.93	5.69	0.75		**0.01**^{d}	**0.04**^{‡,g}**;0.02**$^{¥,g}$
26	3	controls	20	20.25	17.86	13.09	33.56	14.82	25.62	6.43	1.44	0.99^{b}	19.29	19.17	11.55	27.29	14.89	24.08	5.04	1.13	0.86^{a}	20.86	21.43	12.55	31.37	15.14	25.21	5.96	1.33	0.08^{a}	0.39^{d}	
		cases	57	21.16	20.59	9.05	101.00	15.19	23.99	12.06	1.60		19.05	18.90	10.43	36.29	15.31	22.10	4.98	0.66		18.28	18.52	5.31	31.47	14.50	21.47	5.37	0.71		**0.048**^{d}	**0.04**$^{¥,g}$
25	4	controls	20	8.68	8.50	0.00	15.43	7.20	9.84	3.14	0.70	0.96^{b}	8.56	8.43	5.22	13.25	6.61	10.19	2.25	0.50	0.24^{b}	7.80	8.78	0.00	14.43	5.27	10.99	4.84	1.08	0.71^{b}	0.62^{c}	
		cases	57	9.70	8.77	0.00	101.00	7.07	10.55	12.78	1.69		7.39	7.91	0.00	13.17	6.09	9.47	3.31	0.44		7.78	8.18	0.00	15.19	6.55	9.52	3.20	0.42		0.16^{d}	
24	5	controls	20	16.89	15.65	10.69	26.74	11.76	21.85	5.65	1.26	0.68^{b}	16.36	16.63	6.97	25.31	12.74	19.02	4.79	1.07	0.81^{a}	18.41	18.13	9.37	27.41	13.88	22.66	5.07	1.13	0.06^{a}	0.35^{d}	
		cases	57	18.67	16.42	9.15	101.00	13.07	21.33	12.20	1.62		16.07	16.51	7.60	30.81	13.35	18.22	4.71	0.62		16.21	15.77	8.12	26.00	13.27	18.07	4.25	0.56		0.62^{d}	
23	6	controls	20	6.11	5.87	3.83	9.18	4.92	7.44	1.62	0.36	0.74^{b}	6.04	5.67	3.12	11.60	4.84	6.59	1.89	0.42	0.99^{b}	6.53	6.42	3.82	11.06	5.72	7.09	1.65	0.37	0.33^{a}	0.09^{d}	
		cases	57	6.53	6.19	3.39	24.75	5.00	7.53	2.98	0.40		5.89	5.92	0.00	11.92	4.79	6.94	1.88	0.25		6.14	6.23	3.45	10.56	4.95	6.79	1.49	0.20		0.18^{d}	
22	7	controls	20	11.86	10.83	7.69	17.03	9.71	14.63	2.78	0.62	0.88^{b}	11.10	11.07	5.10	17.25	9.49	13.28	2.76	0.62	0.85^{b}	11.85	12.02	7.00	17.27	9.38	13.82	2.88	0.64	0.58^{a}	0.26^{c}	
		cases	57	12.27	10.88	5.69	44.40	9.62	14.24	5.53	0.73		11.61	11.48	6.38	25.14	9.22	12.99	3.38	0.45		11.45	11.45	6.19	18.80	9.22	13.21	2.77	0.37		0.42^{d}	
21	8	controls	20	8.43	7.62	5.12	14.04	6.50	10.25	2.43	0.54	0.81^{b}	8.00	8.25	4.62	12.85	6.51	9.05	2.22	0.50	0.95^{b}	8.22	7.54	5.38	13.03	6.72	9.68	2.12	0.47	0.98^{b}	0.61^{c}	
		cases	57	8.96	8.30	4.64	34.54	6.29	10.28	4.29	0.57		8.15	7.79	4.77	16.96	6.30	9.37	2.41	0.32		8.19	7.96	4.74	13.47	6.69	9.80	2.12	0.28		0.69^{d}	
20	9	controls	20	9.36	8.92	6.40	12.99	7.53	11.43	2.22	0.50	0.71^{b}	8.88	9.92	4.30	14.94	5.90	10.57	2.91	0.65	0.76^{a}	9.31	9.44	5.41	13.64	7.75	10.74	2.09	0.47	0.83^{a}	0.54^{c}	
		cases	57	9.84	9.46	4.41	32.89	7.78	11.21	3.93	0.52		9.09	9.05	5.06	15.66	7.23	10.33	2.36	0.31		9.43	9.27	5.45	14.70	8.03	10.58	2.04	0.27		0.34^{d}	
19	10	controls	20	8.87	7.77	4.46	14.51	7.02	11.27	2.78	0.62	0.88^{b}	8.57	7.89	5.67	13.50	6.54	10.01	2.46	0.55	0.82^{a}	9.12	9.02	4.95	14.08	7.17	10.93	2.43	0.54	0.68^{b}	0.42^{c}	
		cases	57	9.28	8.75	4.34	29.92	6.69	11.65	3.95	0.52		8.75	8.55	0.00	18.73	6.76	10.90	3.24	0.43		8.97	8.82	4.58	14.71	6.93	10.81	2.70	0.36		0.50^{d}	
18	11	controls	20	16.64	18.04	7.55	26.45	12.16	20.54	5.44	1.22	0.60^{b}	16.28	15.18	7.59	26.94	13.40	20.36	4.85	1.08	0.99^{b}	15.60	14.48	9.38	24.47	12.69	18.79	4.21	0.94	0.55^{a}	0.32^{c}	
		cases	57	16.94	16.49	7.14	59.15	11.67	19.47	7.59	1.01		16.77	16.13	7.23	51.43	12.21	20.55	6.85	0.91		16.37	15.97	6.71	30.65	13.17	19.16	5.12	0.68		0.88^{d}	
17	12	controls	20	7.92	6.67	4.55	12.76	5.62	11.04	2.84	0.63	0.77^{b}	8.06	7.80	4.57	14.15	5.87	9.43	2.46	0.55	0.76^{a}	8.57	8.75	4.91	13.91	6.22	10.24	2.66	0.60	0.61^{a}	0.21^{d}	
		cases	57	8.47	8.28	4.15	23.60	5.83	10.63	3.28	0.43		7.86	7.76	0.00	13.11	5.84	9.78	2.56	0.34		8.25	8.29	4.24	13.41	6.42	9.65	2.31	0.31		0.56^{d}	
16	13	controls	20	12.81	10.62	7.44	19.35	9.39	16.93	4.33	0.97	0.93^{b}	12.49	12.57	6.97	19.60	8.76	15.93	3.86	0.86	0.77^{a}	13.33	12.63	6.25	21.92	10.94	16.07	3.78	0.85	0.42^{b}	0.21^{d}	
		cases	57	13.45	12.97	4.30	48.83	8.23	16.47	6.54	0.87		12.17	12.02	0.00	22.00	8.88	15.45	4.44	0.59		12.74	12.51	6.32	26.44	9.35	15.27	4.08	0.54		0.31^{d}	
15	14	controls	20	32.12	28.46	12.10	72.07	21.50	38.03	15.36	3.43	0.41^{b}	30.44	31.89	12.61	52.18	21.25	39.56	11.19	2.50	0.72^{a}	36.00	34.26	12.32	68.21	24.22	47.19	15.08	3.37	0.07^{a}	0.09^{c}	
		cases	57	34.98	35.71	10.34	100.00	20.90	42.50	15.95	2.11		31.61	32.52	7.88	68.11	22.28	39.51	12.69	1.68		29.87	29.75	8.78	55.45	19.69	37.76	11.78	1.56		**0.005**^{d}	**0.003**$^{¥,g}$
14	15	controls	20	25.51	23.60	8.79	47.79	18.82	30.61	10.15	2.27	0.47^{b}	24.65	26.04	9.95	40.70	19.82	29.89	8.52	1.91	0.59^{a}	28.15	28.24	13.05	52.77	20.45	36.23	10.24	2.29	0.36^{a}	0.10^{c}	
		cases	57	28.15	26.72	9.41	97.81	16.31	33.86	14.05	1.86		25.96	25.93	9.31	52.17	18.41	31.14	9.61	1.27		25.69	25.88	9.26	53.36	16.95	30.98	10.40	1.38		0.42^{d}	
13	16	controls	20	11.17	10.26	4.93	17.53	8.14	15.20	4.03	0.90	0.73^{b}	11.24	12.36	5.10	16.88	6.67	15.02	4.14	0.93	0.77^{b}	12.00	11.80	5.18	18.07	9.21	14.30	3.55	0.79	0.64^{a}	0.21^{d}	
		cases	57	12.23	11.44	4.63	41.24	7.64	15.14	5.82	0.77		11.78	11.73	5.51	28.03	8.19	14.49	4.35	0.58		11.52	12.05	4.71	21.29	8.07	14.46	4.05	0.54		0.88^{d}	
12	17	controls	20	17.14	15.15	6.84	31.70	11.17	22.26	8.04	1.80	0.55^{b}	16.86	17.11	6.77	28.22	13.03	20.68	6.29	1.41	0.74^{a}	18.98	19.02	9.23	30.84	13.18	26.03	6.99	1.56	0.20^{b}	0.70^{d}	
		cases	57	18.64	17.85	5.46	65.79	11.44	22.62	9.73	1.29		16.30	16.66	3.92	38.82	10.68	20.95	6.73	0.89		16.92	16.94	7.14	36.27	10.54	21.54	7.09	0.94		0.16^{d}	
11	18	controls	20	22.37	18.90	11.19	43.90	16.80	28.56	9.35	2.09	0.61^{b}	19.52	18.78	5.91	34.60	14.14	24.96	8.02	1.79	0.89^{b}	23.32	20.61	9.64	37.95	17.18	32.78	9.21	2.06	0.14^{b}	**0.02**^{d}	**0,02**$^{£,g}$
		cases	57	21.73	19.14	7.65	81.70	13.25	25.76	12.40	1.64		19.36	18.58	6.33	56.05	13.79	21.98	8.64	1.14		20.01	18.35	8.61	40.34	14.00	25.24	7.98	1.06		0.37^{d}	
10	19	controls	20	16.04	14.31	4.85	29.29	10.93	22.15	7.12	1.59	0.99^{b}	14.45	14.15	6.38	22.73	9.99	18.48	5.22	1.17	0.97^{b}	16.33	14.19	0.00	31.60	11.50	22.63	7.51	1.68	0.54^{a}	0.28^{c}	
		cases	57	16.60	14.65	0.00	63.72	10.45	21.10	9.69	1.28		14.71	14.03	5.45	34.25	11.10	16.74	5.86	0.78		15.22	14.08	0.00	32.52	10.16	18.39	6.66	0.88		0.23^{d}	
9	20	controls	20	7.15	6.76	3.40	13.88	4.73	7.81	3.07	0.69	0.93^{b}	6.26	5.91	3.09	10.43	4.40	7.81	2.09	0.47	0.83^{a}	7.57	7.70	3.11	12.52	5.45	9.46	2.76	0.62	0.23^{i}	0.09^{d}	
		cases	57	7.02	6.73	3.28	13.53	4.89	8.82	2.47	0.33		6.38	6.37	0.00	12.52	5.08	7.59	2.21	0.29		6.76	6.61	2.71	12.61	5.57	7.65	1.80	0.24		0.50^{d}	
8	21	controls	20	9.98	8.38	5.21	15.98	7.03	13.87	3.76	0.84	0.40^{b}	9.75	9.23	4.12	16.51	7.34	12.34	3.36	0.75	0.56^{b}	10.81	9.71	5.89	17.35	8.11	13.75	3.48	0.78	0.22^{b}	0.14^{d}	
		cases	57	9.29	8.74	4.09	21.47	6.48	11.26	3.78	0.50		11.14	8.61	4.71	101.00	6.84	10.84	12.92	1.71		9.77	9.28	4.32	18.92	7.20	11.86	3.43	0.45		0.43^{d}	
7	22	controls	20	5.88	5.33	3.00	10.56	4.24	7.28	2.12	0.47	0.95^{b}	6.07	5.80	3.80	13.19	4.93	6.74	1.99	0.45	0.19^{b}	6.17	5.49	3.69	9.75	4.71	7.68	1.85	0.41	0.16^{b}	0.70^{d}	
		cases	57	5.69	5.40	1.84	11.63	4.59	6.48	1.86	0.25		7.15	5.15	2.92	101.00	4.23	6.47	12.79	1.69		5.47	5.48	2.88	8.82	4.24	6.70	1.49	0.20		0.24^{d}	
6	23	controls	20	10.70	9.74	5.90	18.13	8.08	14.15	3.64	0.81	0.40^{b}	10.13	9.89	4.17	16.81	7.45	12.20	3.51	0.78	0.37^{b}	10.97	11.49	5.62	18.11	7.41	13.24	3.54	0.79	0.25^{b}	0.36^{c}	
		cases	57	9.98	9.84	4.04	21.77	6.83	12.29	3.76	0.50		11.11	8.64	5.26	101.00	6.87	11.64	12.67	1.68		9.90	9.51	5.21	16.45	7.60	11.99	3.00	0.40		0.40^{d}	

Legend: ^{A} – Major curve ≤ 70° vs >70° group p-value; ^{B} – concave vs. convex vs. superficial; ^{a} – t-Student test; ^{b} – Mann-Whitney U test; ^{c} - Repeated measures ANOVA test; ^{d} – Friedman rank test for repeated measures; ^{e} - Repeated measures ANOVA test with Greenhouse-Geisser correction; ^{f} – Tukey HSD post-hoc test ^{g} - Dunn Bonferroni-Hochberg post-hoc test; ^{h} – Benjamini-Hochberg post-hoc test; ; ^{i} – t-Student test with separate variance estimates; ^{j} – Fisher LSD post-hoc test ‡ - convex vs. concave; $^{¥}$ - convex vs. superficial; $^{£}$ - concave vs. superficial; Statistically significant differences are indicated in bold.

Table S3. Methylation level at *LBX1* DNA forward strand promoter region in patients subgroups with major curve ≤ 70° and > 70°, in deep paravertebral muscles and superficial muscles.

CpG	Group	N	Convex									Concave									Superficial									*p*-value[B]	Post-hoc
			M	Me	Min	Max	Q1	Q3	SD	SE	*p*-value[A]	M	Me	Min	Max	Q1	Q3	SD	SE	*p*-value[A]	M	Me	Min	Max	Q1	Q3	SD	SE	*p*-value[A]		
1	Major curve ≤ 70°	28	12.39	12.34	5.61	20.53	9.71	14.73	3.76	0.71	0.22[a]	12.43	12.06	6.46	24.08	8.94	14.87	4.31	0.82	0.06[a]	13.28	13.00	6.37	20.36	10.19	16.95	4.23	0.80	**0.047[a]**	0.30[e]	
	Major curve >70°	29	13.64	13.56	6.36	21.64	10.72	16.70	3.88	0.72		14.36	13.46	7.62	21.30	12.08	16.64	3.45	0.64		15.55	15.23	7.89	25.21	12.98	18.83	4.18	0.78		**0.03[c]**	**0.02[¥,f]**
2	Major curve ≤ 70°	28	12.25	11.88	4.13	19.99	10.07	15.51	3.81	0.72	0.29[a]	12.15	11.93	5.84	22.59	9.95	14.27	3.75	0.71	**0.02[a]**	13.00	12.96	5.87	19.74	10.25	16.33	3.97	0.75	0.06[a]	0.34[c]	
	Major curve >70°	29	13.38	13.74	6.11	20.24	10.01	16.66	4.14	0.77		14.44	14.21	8.10	22.02	12.97	15.78	3.40	0.63		15.16	15.08	7.26	25.51	11.87	18.34	4.45	0.83		**0.04[c]**	**0.03[¥,f]**
3	Major curve ≤ 70°	28	14.69	15.13	6.21	22.54	11.53	16.95	4.02	0.76	0.24[a]	14.68	14.80	7.79	21.44	11.38	17.84	3.95	0.75	0.20[a]	16.24	16.77	8.30	22.50	13.05	19.96	4.15	0.78	0.54[a]	**0.03[c]**	**0.03[¥,j];0.03[£,j]**
	Major curve >70°	29	16.04	15.79	6.36	26.85	14.44	19.31	4.43	0.82		15.96	15.56	8.70	22.29	13.82	18.75	3.48	0.65		16.89	16.50	8.93	27.73	14.74	18.10	3.86	0.72		0.32[c]	
4	Major curve ≤ 70°	28	17.01	17.38	7.43	26.23	13.09	20.31	4.67	0.88	0.29[a]	17.32	18.01	7.76	26.32	13.73	19.56	4.89	0.92	0.16[a]	18.35	18.95	7.44	28.34	13.99	22.38	5.28	1.00	0.14[a]	0.15[c]	
	Major curve >70°	29	18.38	18.29	9.51	27.79	14.65	22.53	5.05	0.94		19.10	18.94	9.68	26.89	16.16	22.10	4.45	0.83		20.38	19.50	11.77	30.61	17.05	23.32	5.00	0.93		**0.02[c]**	**0.01[¥,f]**
5	Major curve ≤ 70°	28	16.11	16.62	6.23	24.93	13.08	18.54	4.15	0.78	0.50[a]	16.05	16.18	7.68	25.25	12.89	18.77	4.35	0.82	0.29[a]	17.11	17.24	7.84	25.90	14.02	20.44	4.58	0.87	0.24[a]	0.21[c]	
	Major curve >70°	29	16.89	16.76	7.34	26.89	14.61	20.51	4.54	0.84		17.23	16.67	10.40	24.63	14.93	19.35	4.08	0.76		18.59	17.76	10.95	31.97	15.14	21.27	4.76	0.88		0.08[c]	
6	Major curve ≤ 70°	28	16.56	16.32	5.34	36.05	12.33	19.29	6.06	1.15	0.27[a]	18.50	17.19	6.76	42.66	12.19	23.55	8.25	1.56	0.77[i]	18.61	18.55	9.15	32.43	14.19	21.41	6.22	1.18	0.83[a]	0.15[e]	
	Major curve >70°	29	18.40	18.44	7.05	36.22	15.03	20.99	6.25	1.16		17.98	16.67	9.39	29.25	15.09	21.43	5.04	0.94		18.91	19.18	9.11	26.38	16.05	22.02	5.02	0.93		0.59[c]	
7	Major curve ≤ 70°	28	16.04	14.92	4.84	24.52	12.28	20.08	5.13	0.97	0.25[a]	16.62	16.14	6.25	28.69	11.54	19.96	6.00	1.13	0.23[a]	17.52	18.34	7.76	26.63	13.44	21.71	5.35	1.01	0.20[a]	0.19[c]	
	Major curve >70°	29	17.62	17.29	8.09	28.30	13.67	22.18	5.15	0.96		18.41	18.62	7.56	28.58	15.20	20.76	5.03	0.93		19.33	18.03	11.00	29.17	16.38	23.48	5.15	0.96		0.09[c]	
8	Major curve ≤ 70°	28	22.79	20.32	7.80	35.49	17.74	29.23	7.26	1.37	0.37[a]	23.53	22.75	8.00	42.34	16.37	28.08	8.79	1.66	0.29[a]	24.60	25.12	11.57	41.32	16.77	30.04	7.98	1.51	0.11[a]	0.18[e]	
	Major curve >70°	29	24.56	23.79	10.02	38.27	18.11	32.02	7.75	1.44		25.78	25.86	13.58	39.02	21.34	27.66	7.05	1.31		28.01	26.88	14.37	42.44	21.84	34.54	7.76	1.44		**0.01[c]**	**0.008[¥,f]**
9	Major curve ≤ 70°	28	16.82	15.80	6.21	25.96	13.43	20.87	4.80	0.91	0.25[a]	17.42	17.75	8.40	28.05	13.01	21.28	5.29	1.00	0.25[a]	18.28	17.76	9.67	29.70	14.63	22.55	5.18	0.98	0.16[a]	0.14[c]	
	Major curve >70°	29	18.35	17.78	8.65	29.09	14.47	22.78	5.08	0.94		18.94	18.24	10.19	26.86	16.20	23.67	4.69	0.87		20.17	19.34	12.47	29.30	18.26	22.88	4.75	0.88		0.05[c]	
10	Major curve ≤ 70°	28	23.95	23.77	8.36	37.01	19.38	27.18	6.65	1.26	0.76[a]	25.11	24.81	9.86	43.54	19.42	31.80	7.55	1.43	0.86[a]	25.66	25.69	14.46	37.81	20.21	31.31	6.35	1.20	0.64[a]	0.23[c]	
	Major curve >70°	29	24.49	25.84	9.80	36.46	19.05	29.71	6.93	1.29		25.45	25.20	14.34	36.32	21.43	30.47	6.18	1.15		26.46	24.85	16.04	38.61	23.01	31.03	6.37	1.18		0.17[c]	
11	Major curve ≤ 70°	28	20.85	21.59	9.40	31.10	17.56	23.70	4.80	0.91	0.31[a]	21.18	20.19	9.79	35.83	16.80	25.86	6.08	1.15	0.19[a]	21.83	21.51	14.73	32.88	18.09	25.57	4.72	0.89	0.12[a]	0.40[e]	
	Major curve >70°	29	22.23	21.85	10.85	32.92	18.48	26.69	5.30	0.98		23.15	22.14	14.14	32.96	20.25	27.04	5.06	0.94		23.84	22.39	15.14	32.06	19.98	28.12	5.03	0.93		0.09[c]	
12	Major curve ≤ 70°	28	20.91	20.48	10.10	31.03	17.16	24.84	5.02	0.95	0.48[a]	21.66	21.91	11.92	33.43	17.07	25.64	5.67	1.07	0.38[a]	22.23	23.07	13.49	31.34	18.39	25.27	4.79	0.90	0.24[a]	0.24[e]	
	Major curve >70°	29	21.91	22.55	12.21	31.11	18.27	25.93	5.48	1.02		22.92	22.66	12.46	32.43	20.71	26.36	5.12	0.95		23.77	22.55	15.50	32.98	19.66	27.91	5.06	0.94		**0.04[c]**	**0.03[¥,f]**
13	Major curve ≤ 70°	28	22.51	22.14	12.45	33.45	18.98	25.81	5.00	0.94	0.24[a]	22.65	21.75	14.07	32.37	18.61	27.07	5.04	0.95	0.08[a]	23.66	23.10	15.17	33.67	20.12	26.73	4.88	0.92	0.13[a]	0.19[e]	
	Major curve >70°	29	24.15	23.12	13.61	32.75	19.70	29.56	5.48	1.02		25.07	25.89	16.79	35.28	19.85	28.58	5.27	0.98		25.78	25.72	15.55	34.81	21.13	30.71	5.71	1.06		0.12[c]	
14	Major curve ≤ 70°	28	7.88	7.13	4.20	14.31	6.30	8.74	2.35	0.44	**0.04**[b]	8.01	7.58	4.88	13.26	5.73	9.86	2.59	0.49	**0.03**[b]	8.14	7.83	4.59	16.86	6.95	8.71	2.65	0.50	**0.001**[b]	0.33[d]	
	Major curve >70°	29	9.47	8.34	4.85	16.44	7.45	11.67	3.01	0.56		9.62	8.82	6.09	16.56	7.30	12.14	2.94	0.55		10.73	9.86	5.01	15.35	8.42	13.94	3.34	0.62		0.05[d]	
15	Major curve ≤ 70°	28	27.49	25.73	17.23	40.82	21.20	32.62	7.72	1.46	0.15[b]	28.11	27.41	16.66	45.12	20.21	33.37	8.43	1.59	**0.03**[b]	28.97	27.91	17.56	42.85	21.41	34.62	7.95	1.50	**0.02**[b]	0.78[d]	
	Major curve >70°	29	30.65	29.81	16.86	44.48	24.69	37.93	7.92	1.47		32.34	33.46	19.00	46.72	26.60	37.16	7.32	1.36		33.99	34.94	19.90	44.41	28.49	40.39	7.57	1.40		**0.004[c]**	**0.001[¥,f]**
16	Major curve ≤ 70°	28	23.22	22.37	12.61	37.59	19.37	25.28	5.55	1.05	0.07[b]	23.79	23.66	14.37	36.02	19.85	26.38	5.52	1.04	**0.02[a]**	24.36	23.54	15.29	36.80	20.79	27.76	5.33	1.01	**0.01[a]**	0.06[d]	
	Major curve >70°	29	26.73	25.72	15.00	37.26	21.62	33.96	7.06	1.31		27.89	27.00	16.26	39.63	23.40	33.41	6.84	1.27		28.41	28.48	17.28	38.31	22.20	33.81	6.57	1.22		0.64[d]	
17	Major curve ≤ 70°	28	11.77	13.14	4.97	17.59	7.61	15.16	4.01	0.76	0.08[b]	11.55	12.97	2.33	18.56	7.01	14.62	4.34	0.82	**0.01**[a]	11.98	12.54	2.48	18.68	7.69	14.92	4.37	0.83	**0.001**[b]	0.63[d]	
	Major curve >70°	29	13.61	14.37	6.72	18.49	11.37	16.39	3.38	0.63		14.07	14.49	6.64	18.32	11.81	16.23	3.01	0.56		14.27	14.79	6.20	18.83	13.62	16.68	3.13	0.58		0.22[e]	
18	Major curve ≤ 70°	28	8.14	7.71	4.40	14.72	6.09	8.49	2.66	0.50	**0.001**[b]	8.62	7.89	4.52	25.58	5.87	8.92	4.27	0.81	**0.005**[b]	8.74	8.42	4.53	16.58	7.19	9.98	2.65	0.50	**0.03**[b]	**0.02[d]**	**0.046[¥,g];0.03[£,g]**
	Major curve >70°	29	10.38	8.98	5.93	15.96	7.86	13.35	3.11	0.58		10.68	10.52	5.72	15.38	7.97	13.19	2.99	0.56		10.63	10.82	5.34	14.43	7.98	13.66	3.05	0.57		0.52[d]	
19	Major curve ≤ 70°	28	8.98	8.81	5.12	14.33	6.64	10.93	2.76	0.52	**0.04**[b]	8.72	8.14	2.27	15.76	6.37	10.40	3.08	0.58	**0.01**[b]	9.19	8.63	5.79	16.15	6.77	11.55	2.77	0.52	**0.02**[b]	0.25[d]	
	Major curve >70°	29	11.04	10.63	5.62	25.49	8.18	13.02	4.01	0.74		10.91	11.33	6.31	14.91	7.69	13.23	2.75	0.51		10.82	10.85	5.60	14.86	8.27	13.20	2.65	0.49		0.42[d]	
20	Major curve ≤ 70°	28	16.76	17.37	9.03	21.78	14.15	18.79	3.19	0.60	0.35[a]	17.93	17.73	10.92	27.68	14.70	20.36	3.86	0.73	0.69[a]	18.49	17.94	11.35	24.53	15.94	21.60	3.29	0.62	0.77[a]	0.05[c]	
	Major curve >70°	29	17.66	18.13	8.98	24.14	14.03	20.97	4.01	0.74		18.29	18.65	12.86	24.84	16.09	20.37	2.87	0.53		18.76	19.05	10.07	24.30	17.08	21.89	3.64	0.68		0.08[c]	
21	Major curve ≤ 70°	28	35.40	29.00	19.54	59.24	26.22	51.03	13.23	2.50	0.12[b]	35.10	29.10	21.32	59.15	24.59	52.18	13.49	2.55	0.91[b]	36.18	30.15	21.04	68.82	27.20	49.46	13.92	2.63	0.17[b]	0.10[d]	
	Major curve >70°	29	29.37	27.69	19.49	55.95	24.82	32.11	8.20	1.52		30.45	29.41	21.98	57.62	26.25	31.31	8.02	1.49		30.42	29.35	21.71	59.70	26.43	32.59	8.00	1.49		0.11[d]	
22	Major curve ≤ 70°	28	16.61	16.61	9.59	24.84	13.58	19.32	4.09	0.77	0.45[a]	17.16	17.01	9.40	24.94	15.46	19.69	3.96	0.75	0.23[a]	17.08	16.80	0.00	32.34	14.66	20.48	5.60	1.06	0.09[b]	0.25[d]	
	Major curve >70°	29	17.45	17.04	11.15	25.70	14.05	21.15	4.20	0.78		18.40	17.69	9.16	25.42	16.88	20.94	3.75	0.70		19.20	19.42	11.42	30.16	16.71	22.26	4.43	0.82		**0.01[c]**	**0.03[¥,g]**
23	Major curve ≤ 70°	28	16.19	16.18	7.68	29.73	11.77	18.94	5.46	1.03	0.79[b]	14.92	15.37	0.00	23.16	11.71	18.70	5.29	1.00	0.06[b]	16.17	15.81	8.24	24.63	11.86	20.00	5.06	0.96	0.82[b]	0.26[c]	
	Major curve >70°	29	17.43	16.43	0.00	57.38	11.53	20.39	9.72	1.80		19.34	17.37	9.51	74.72	14.83	20.88	11.64	2.16		16.34	17.04	0.00	23.90	12.63	21.09	5.50	1.02		0.06[d]	

Legend: [A] – Major curve ≤ 70° vs. >70° group p-value; [B] – concave vs. convex vs. superficial; [a] – t-Student test; [b] – Mann-Whitney U test; [c] - Repeated measures ANOVA test; [d] – Friedman rank test for repeated measures; [e] - Repeated measures ANOVA test with Greenhouse-Geisser correction; [f] – Tukey HSD post-hoc test [g] - Dunn Bonferroni-Hochberg post-hoc test; [h] – Benjamini-Hochberg post-hoc test; ; [i] – t-Student test with separate variance estimates; [j] – Fisher LSD post-hoc test [‡] - convex vs. concave; [¥] - convex vs. superficial; [£] - concave vs. superficial; Statistically significant differences are indicated in bold.

Table S4. Methylation level at *LBX1* DNA reverse strand promoter region in patients subgroup with major curve ≤ 70° and > 70°, in deep paravertebral muscles and superficial muscles.

CpG	Group	N	Convex									Concave									Superficial									*p*-value[B]	Post-hoc
			M	Me	Min	Max	Q1	Q3	SD	SE	p-value[A]	M	Me	Min	Max	Q1	Q3	SD	SE	p-value[A]	M	Me	Min	Max	Q1	Q3	SD	SE	p-value[A]		
1	Major curve ≤ 70°	28	31.91	33.60	13.61	51.89	25.89	37.94	10.37	1.96	0.14[b]	30.64	31.52	0.00	57.41	19.48	40.35	12.91	2.44	0.52[b]	29.86	30.54	13.50	52.03	21.12	34.43	10.34	1.95	0.23[a]	0.40[c]	
	Major curve >70°	29	38.59	34.68	12.62	101.00	30.31	45.87	17.20	3.19		31.99	32.58	0.00	47.33	30.24	36.43	10.25	1.90		33.36	30.97	9.98	54.76	26.40	40.54	11.54	2.14		**0.01[d]**	**0.03[¥,g]; 0.03[‡,g]**
2	Major curve ≤ 70°	28	23.71	24.46	13.82	38.04	18.86	27.91	6.20	1.17	0.07[b]	22.73	22.81	12.52	38.17	15.85	28.14	6.96	1.32	0.36[b]	22.16	23.79	11.09	31.07	18.54	26.28	5.50	1.04	0.22[a]	0.94[c]	
	Major curve >70°	29	29.03	26.41	14.13	101.00	22.21	30.61	15.28	2.84		24.34	23.27	15.78	43.84	20.38	27.53	5.55	1.03		24.04	23.79	12.27	38.16	20.75	28.47	5.81	1.08		**0.02[d]**	**0.03[‡,g]**
3	Major curve ≤ 70°	28	18.44	18.58	9.05	27.71	13.37	22.47	5.32	1.01	0.06[b]	18.32	18.71	10.43	27.01	14.48	22.55	4.93	0.93	0.35[b]	17.40	17.63	5.31	28.62	14.30	20.24	5.09	0.96	0.23[a]	0.42[c]	
	Major curve >70°	29	23.79	21.73	9.29	101.00	17.69	24.68	15.79	2.93		19.75	18.90	11.36	36.29	16.61	22.10	5.02	0.93		19.13	18.52	9.69	31.47	17.33	23.15	5.59	1.04		0.08[d]	
4	Major curve ≤ 70°	28	7.89	8.54	0.00	12.38	6.75	9.41	2.81	0.53	0.16[b]	6.95	7.80	0.00	10.75	5.79	8.91	2.95	0.56	0.40[b]	6.75	7.67	0.00	12.45	5.11	8.86	3.76	0.71	0.07[b]	0.26[d]	
	Major curve >70°	29	11.44	9.15	0.00	101.00	7.65	11.37	17.68	3.28		7.81	7.91	0.00	13.17	6.19	10.42	3.62	0.67		8.78	8.18	5.02	15.19	7.07	10.05	2.19	0.41		0.45[d]	
5	Major curve ≤ 70°	28	15.98	15.11	9.15	26.96	12.81	18.16	4.90	0.93	**0.04**[b]	15.41	16.17	7.90	25.94	11.22	18.42	4.65	0.88	0.44[b]	15.69	15.41	8.67	26.00	12.49	17.57	4.11	0.78	0.37[a]	0.78[d]	
	Major curve >70°	29	21.27	18.13	10.36	101.00	14.47	23.49	16.13	3.00		16.71	16.52	7.60	30.81	13.77	18.22	4.77	0.89		16.71	16.44	8.12	25.16	14.08	19.66	4.40	0.82		0.17[d]	
6	Major curve ≤ 70°	28	5.82	5.65	3.39	10.87	4.23	6.63	1.76	0.33	**0.03**[b]	5.86	5.72	3.26	11.92	4.66	6.94	1.88	0.36	0.06[b]	6.00	6.03	3.45	8.73	5.09	6.90	1.36	0.26	0.50[a]	0.21[d]	
	Major curve >70°	29	7.22	7.02	3.73	24.75	5.49	7.66	3.71	0.69		5.91	5.92	0.00	10.29	4.85	7.07	1.92	0.36		6.27	6.31	3.85	10.56	4.95	6.79	1.61	0.30		0.19[d]	
7	Major curve ≤ 70°	28	10.70	10.35	5.69	20.08	7.84	12.94	3.52	0.67	**0.02**[b]	11.35	10.82	6.38	25.14	8.56	13.16	3.80	0.72	0.35[b]	11.31	11.50	6.95	16.06	9.07	13.62	2.73	0.52	0.71[a]	0.47[d]	
	Major curve >70°	29	13.78	11.64	8.43	44.40	10.42	15.06	6.66	1.24		11.87	11.77	7.27	20.04	9.96	12.99	2.96	0.55		11.58	11.36	6.19	18.80	9.99	13.21	2.85	0.53		**0.01[d]**	**0.03[¥,g]; 0.03[‡,g]**
8	Major curve ≤ 70°	28	7.77	7.98	5.06	13.19	5.78	8.86	2.28	0.43	**0.03**[b]	7.87	7.68	5.11	12.04	5.90	9.30	2.20	0.42	0.58[b]	8.12	7.84	4.74	12.70	6.40	9.78	2.18	0.41	0.80[a]	0.18[d]	
	Major curve >70°	29	10.10	8.93	4.64	34.54	7.76	11.42	5.40	1.00		8.42	7.79	4.77	16.96	7.01	9.37	2.61	0.48		8.26	7.96	5.29	13.47	6.77	9.80	2.09	0.39		**0.01[d]**	**0.02[¥,g]; 0.04[‡,g]**
9	Major curve ≤ 70°	28	8.74	8.86	4.41	14.54	6.80	10.18	2.28	0.43	**0.02**[b]	9.01	8.68	5.06	15.66	6.74	10.55	2.72	0.51	0.81[b]	9.16	9.15	5.45	12.91	7.89	10.65	1.88	0.36	0.55[a]	0.62[c]	
	Major curve >70°	29	10.90	10.09	5.13	32.89	8.78	12.44	4.84	0.90		9.16	9.05	5.76	13.33	7.52	10.29	2.01	0.37		9.69	9.29	6.70	14.70	8.05	10.27	2.18	0.41		0.08[d]	
10	Major curve ≤ 70°	28	8.26	7.87	4.34	15.39	6.22	9.70	2.73	0.52	0.06[b]	8.15	8.06	0.00	18.73	6.28	10.84	3.83	0.72	0.17[i]	8.75	8.38	4.62	13.14	6.77	11.37	2.68	0.51	0.47[a]	0.17[d]	
	Major curve >70°	29	10.26	9.21	4.89	29.92	7.60	12.09	4.69	0.87		9.34	8.82	5.74	15.57	7.40	10.90	2.49	0.46		9.18	8.84	4.58	14.71	7.90	10.68	2.75	0.51		0.42[d]	
11	Major curve ≤ 70°	28	15.06	13.63	7.14	31.83	11.54	18.69	5.50	1.04	**0.045**[b]	16.74	15.75	7.84	51.43	11.01	20.00	8.60	1.62	0.30[b]	16.35	15.06	8.12	30.65	12.30	19.37	5.72	1.08	0.98[a]	0.33[d]	
	Major curve >70°	29	18.75	17.18	9.59	59.15	15.20	20.34	8.90	1.65		16.80	16.20	7.23	25.66	13.15	20.80	4.74	0.88		16.39	17.07	6.71	27.13	13.75	18.97	4.57	0.85		0.11[d]	
12	Major curve ≤ 70°	28	7.44	6.86	4.15	13.34	5.31	9.21	2.46	0.46	**0.02**[b]	7.55	7.70	0.00	12.86	5.33	9.80	2.82	0.53	0.39[a]	8.10	8.24	4.24	11.86	6.41	9.63	2.15	0.41	0.63[b]	0.17[d]	
	Major curve >70°	29	9.47	9.36	4.61	23.60	6.64	10.80	3.69	0.68		8.15	7.76	4.11	13.11	6.26	9.78	2.30	0.43		8.40	8.29	4.53	13.41	6.47	9.78	2.48	0.46		0.14[d]	
13	Major curve ≤ 70°	28	11.66	11.62	4.30	23.30	7.96	15.22	4.75	0.90	**0.04**[b]	11.42	10.80	0.00	22.00	7.71	15.36	5.09	0.96	0.16[b]	12.24	12.03	6.32	21.59	8.83	15.44	3.96	0.75	0.47[b]	0.43[c]	
	Major curve >70°	29	15.18	15.07	6.71	48.83	11.50	17.04	7.58	1.41		12.89	12.45	8.43	20.79	9.68	15.45	3.64	0.68		13.21	12.51	7.82	26.44	10.33	14.68	4.22	0.78		0.18[d]	
14	Major curve ≤ 70°	28	29.90	33.62	12.34	55.44	17.33	40.35	13.24	2.50	**0.03**[b]	29.34	29.36	7.88	64.10	17.64	40.19	14.28	2.70	0.19[a]	28.67	27.02	11.43	55.45	18.37	37.58	11.98	2.26	0.46[a]	0.78[d]	
	Major curve >70°	29	39.88	39.62	10.34	100.00	31.83	46.62	17.01	3.16		33.80	33.36	15.62	68.11	27.00	39.42	10.75	2.00		31.02	29.78	8.78	54.56	21.38	40.05	11.67	2.17		**<0.001[d]**	**<0.001[¥,g]; 0.04[£,g]**
15	Major curve ≤ 70°	28	23.89	24.13	9.41	47.59	15.72	29.45	10.39	1.96	**0.02**[b]	24.33	24.42	9.31	51.95	15.51	31.56	10.55	1.99	0.21[a]	24.68	21.79	9.89	53.36	15.44	30.88	11.49	2.17	0.32[b]	0.51[d]	
	Major curve >70°	29	32.26	30.48	11.48	97.81	23.27	35.24	15.97	2.97		27.54	26.54	11.61	52.17	22.65	30.70	8.49	1.58		26.67	26.32	9.26	47.95	23.21	33.16	9.32	1.73		**0.03[d]**	**0.03[¥,g]**
16	Major curve ≤ 70°	28	10.77	10.25	5.03	20.42	6.99	14.39	4.38	0.83	**0.047**[b]	11.22	9.65	5.51	28.03	7.34	14.28	5.04	0.95	0.13[b]	11.09	9.56	4.74	21.29	7.44	14.92	4.44	0.84	0.44[a]	0.78[d]	
	Major curve >70°	29	13.65	14.65	4.63	41.24	9.48	15.90	6.70	1.25		12.32	12.34	6.24	21.10	9.66	14.55	3.57	0.66		11.92	12.23	4.71	19.71	9.54	14.30	3.68	0.68		0.39[d]	
17	Major curve ≤ 70°	28	16.27	15.01	6.88	35.58	9.77	21.07	7.41	1.40	0.07[b]	15.12	14.61	3.92	38.82	8.76	20.46	7.44	1.41	0.15[b]	15.44	12.14	7.87	30.76	9.57	18.12	6.66	1.26	0.12[b]	0.37[d]	
	Major curve >70°	29	20.94	18.65	5.46	65.79	15.78	24.40	11.19	2.08		17.43	16.70	6.84	31.82	13.87	21.29	5.87	1.09		18.36	16.94	7.14	36.27	13.65	23.38	7.31	1.36		0.42[d]	
18	Major curve ≤ 70°	28	18.74	17.01	7.65	43.90	10.76	24.49	10.14	1.92	**0.04**[b]	18.45	17.85	6.33	56.05	11.92	21.17	10.01	1.89	0.24[b]	19.25	17.87	9.10	40.34	13.44	22.80	8.08	1.53	0.41[b]	0.65[d]	
	Major curve >70°	29	24.62	22.22	8.77	81.70	17.24	28.40	13.81	2.56		20.24	18.61	9.61	42.26	16.13	23.48	7.14	1.33		20.75	19.07	8.61	37.78	14.02	25.97	7.94	1.47		0.39[d]	
19	Major curve ≤ 70°	28	13.93	13.16	6.16	31.62	8.63	16.73	6.60	1.25	**0.02**[b]	13.99	13.70	5.45	34.25	9.86	16.65	5.91	1.12	0.30[b]	14.19	13.27	0.00	32.52	9.61	16.92	7.01	1.32	0.25[a]	0.96[d]	
	Major curve >70°	29	19.18	16.68	0.00	63.72	13.13	24.13	11.47	2.13		15.41	14.03	6.06	33.19	11.59	17.89	5.83	1.08		16.22	14.37	7.76	31.24	11.06	20.30	6.25	1.16		**0.03[d]**	**0.03[¥,g]**
20	Major curve ≤ 70°	28	6.35	5.73	3.28	12.41	4.71	7.37	2.35	0.44	**0.03**[b]	5.76	5.83	0.00	12.52	4.04	7.43	2.38	0.45	**0.04**[a]	6.52	6.23	4.00	9.14	5.61	7.40	1.30	0.25	0.34[a]	0.17[d]	
	Major curve >70°	29	7.66	7.19	3.95	13.53	5.96	9.37	2.46	0.46		6.99	6.37	4.05	11.76	5.74	8.04	1.87	0.35		6.98	6.67	2.71	12.61	5.47	8.23	2.18	0.40		0.26[d]	
21	Major curve ≤ 70°	28	8.51	6.82	4.63	21.47	5.90	9.31	4.19	0.79	**0.02**[b]	9.48	7.82	4.71	33.17	6.28	10.31	5.69	1.08	0.16[b]	9.33	8.48	4.70	17.56	7.15	11.28	3.13	0.59	0.36[b]	0.16[d]	
	Major curve >70°	29	10.05	10.45	4.09	16.26	7.05	11.80	3.23	0.60		12.74	9.07	5.47	101.00	7.44	11.68	17.24	3.20		10.18	9.41	4.32	18.92	7.41	12.27	3.71	0.69		0.87[d]	
22	Major curve ≤ 70°	28	5.20	4.85	1.84	11.63	4.23	5.45	1.97	0.37	**0.006**[b]	5.41	4.90	2.92	13.74	4.10	5.57	2.24	0.42	0.26[b]	5.32	5.12	3.19	8.20	4.19	6.25	1.44	0.27	0.47[a]	0.90[d]	
	Major curve >70°	29	6.15	6.09	3.11	10.07	5.10	6.98	1.65	0.31		8.84	5.35	3.17	101.00	4.39	6.67	17.79	3.30		5.61	5.82	2.88	8.82	4.25	6.82	1.54	0.29		**0.02[d]**	**0.02[¥,g]**
23	Major curve ≤ 70°	28	8.96	7.62	4.04	21.77	6.13	11.29	3.90	0.74	**0.01**[b]	9.26	7.44	5.26	27.39	6.40	10.86	4.62	0.87	0.08[b]	9.42	8.42	5.58	15.75	7.31	11.55	2.81	0.53	0.19[b]	0.07[d]	
	Major curve >70°	29	10.97	11.01	5.73	18.13	7.95	13.39	3.39	0.63		12.89	9.49	6.09	101.00	7.53	11.81	17.15	3.18		10.36	10.05	5.21	16.45	8.34	12.68	3.15	0.59		0.28[d]	

Legend: [A] – Major curve ≤ 70° vs >70° group p-value; [B] – concave vs. convex vs. superficial; [a] – t-Student test; [b] – Mann-Whitney U test; [c] - Repeated measures ANOVA test; [d] – Friedman rank test for repeated measures; [e] - Repeated measures ANOVA test with Greenhouse-Geisser correction; [f] – Tukey HSD post-hoc test [g] - Dunn Bonferroni-Hochberg post-hoc test; [h] – Benjamini-Hochberg post-hoc test; ; [i] – t-Student test with separate variance estimates; [j] – Fisher LSD post-hoc test [‡] - convex vs. concave; [¥] - convex vs. superficial; [£] - concave vs. superficial; Statistically significant differences are indicated in bold.
