# Supplementary material for: Association of LBX1 Gene Methylation Level with Disease Severity in Patients with Idiopathic Scoliosis: Study on Deep Paravertebral Muscles
Source: Genes (Basel). 2022 Aug 29;13(9):1556. doi: 10.3390/genes13091556 (PMC9498322; doi:10.3390/genes13091556)
Supplement: Supplementary file 1 [file genes-13-01556-s001.zip › genes-1837166-supplementary/Additional file 2.pdf]

Additional file 2

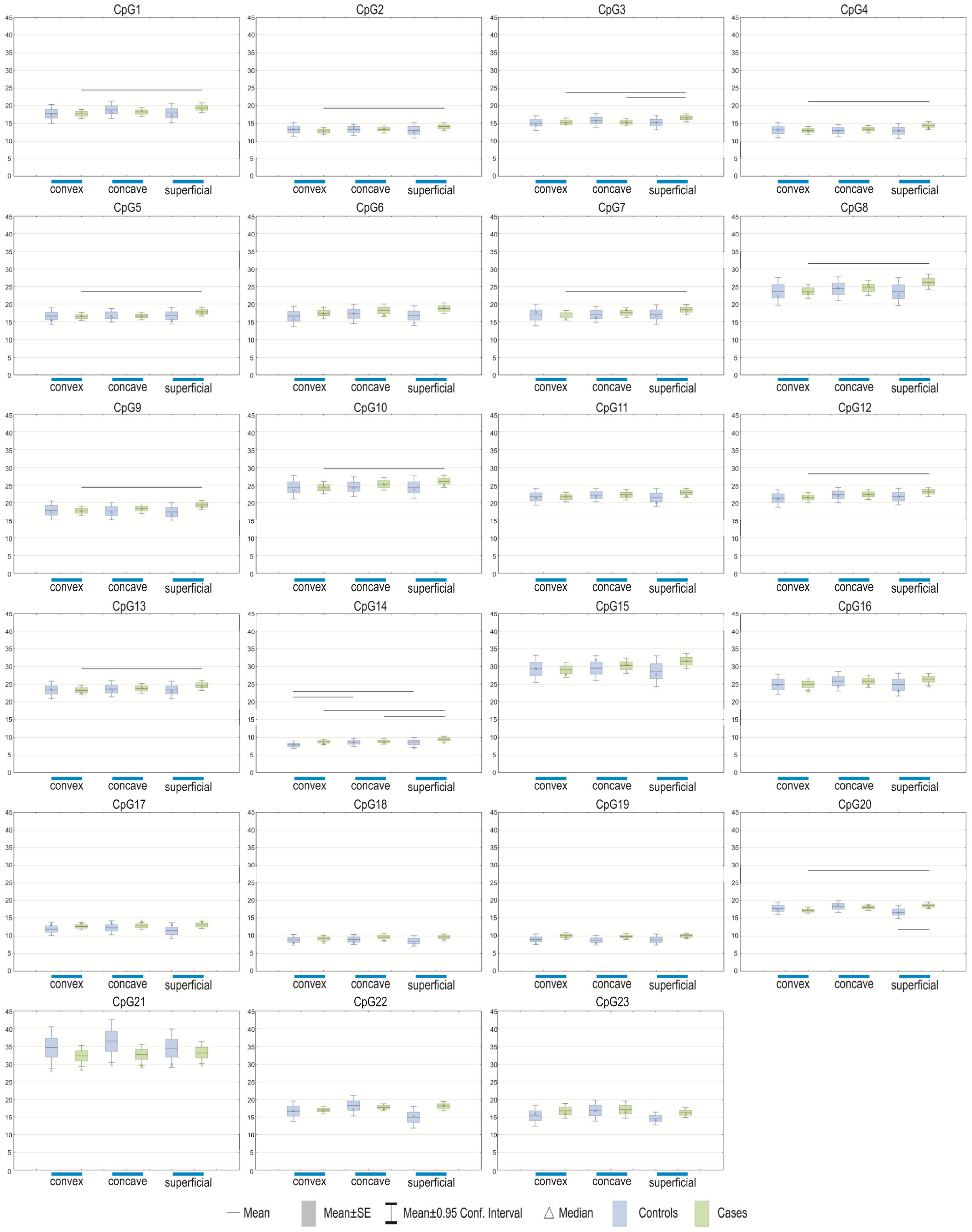

Figure S1. DNA methylation level within *LBX1* forward strand promoter region in deep paravertebral muscles and superficial muscles. Upper solid horizontal lines represent significant differences between muscles, and lower lines show significant differences in case-control study.

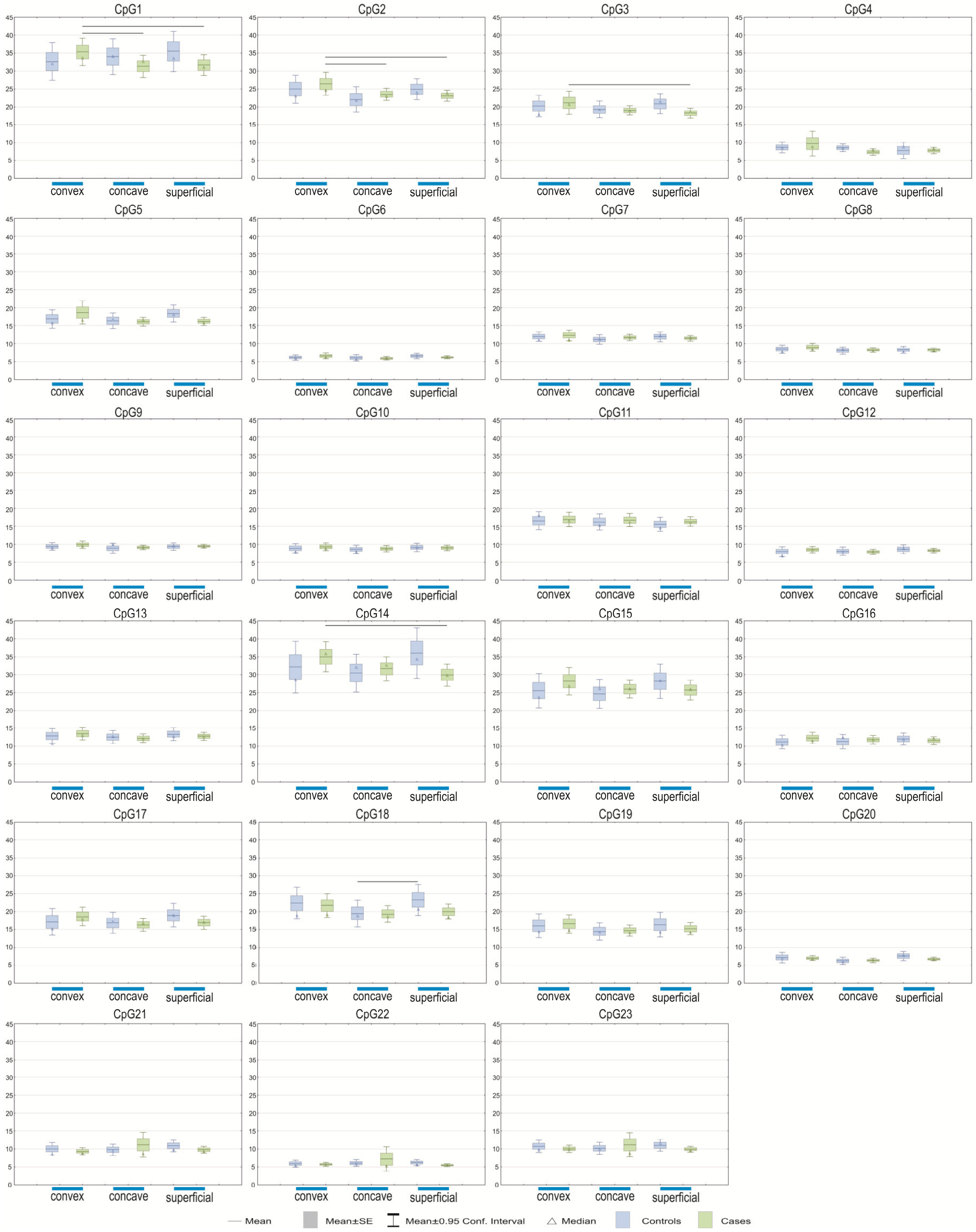

Figure S2. DNA methylation level within *LBX1* reverse strand promoter region in deep paravertebral muscles and superficial muscles. Upper solid horizontal lines represent significant differences between muscles, and lower lines show significant differences in case-control study.

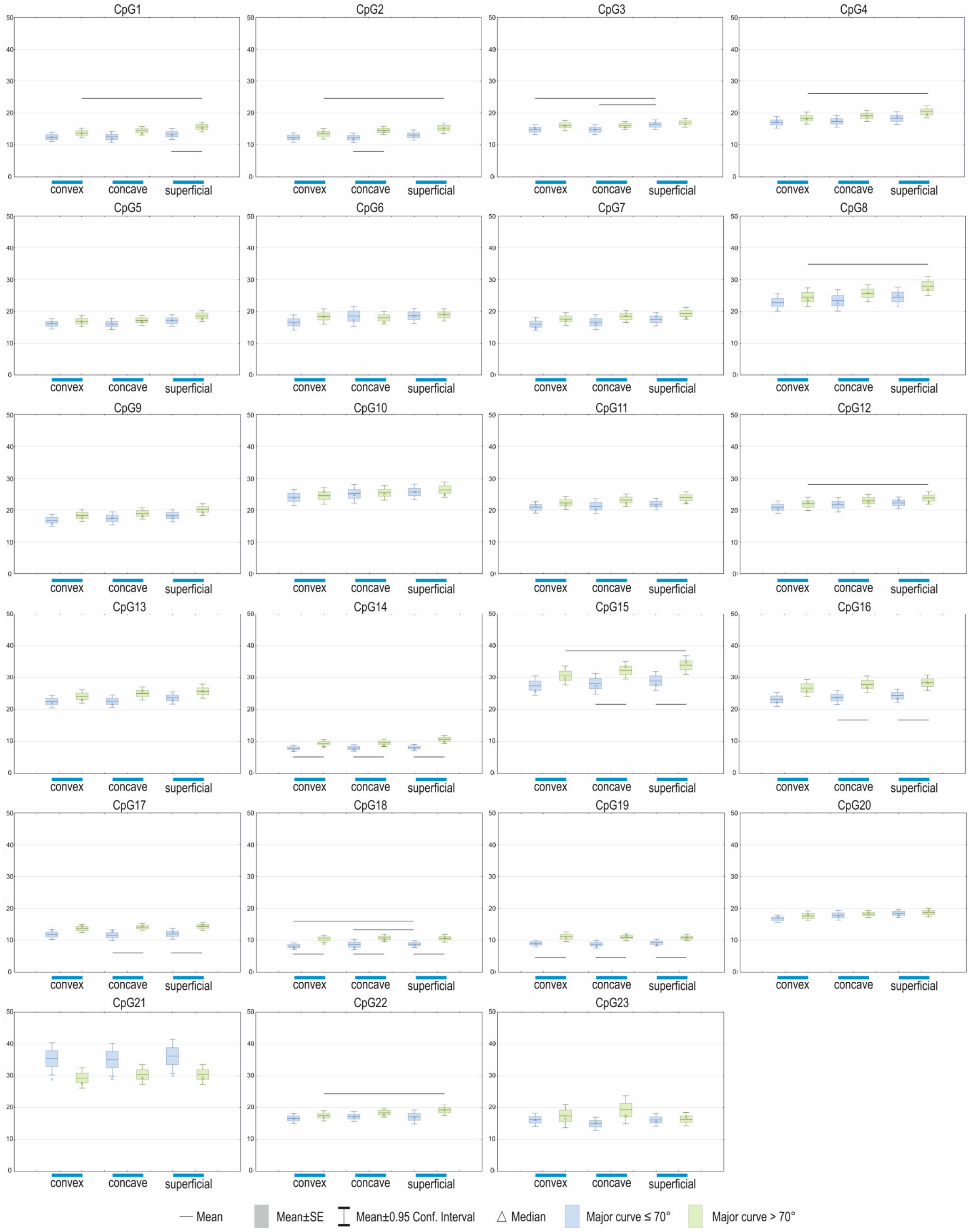

Figure S3. DNA methylation level within *LBX1* forward strand promoter region in deep paravertebral muscles and superficial muscles. Upper solid horizontal lines represent significant differences between muscles in case of patients, and lower lines show significant differences in subgroups divided according to major curve values.

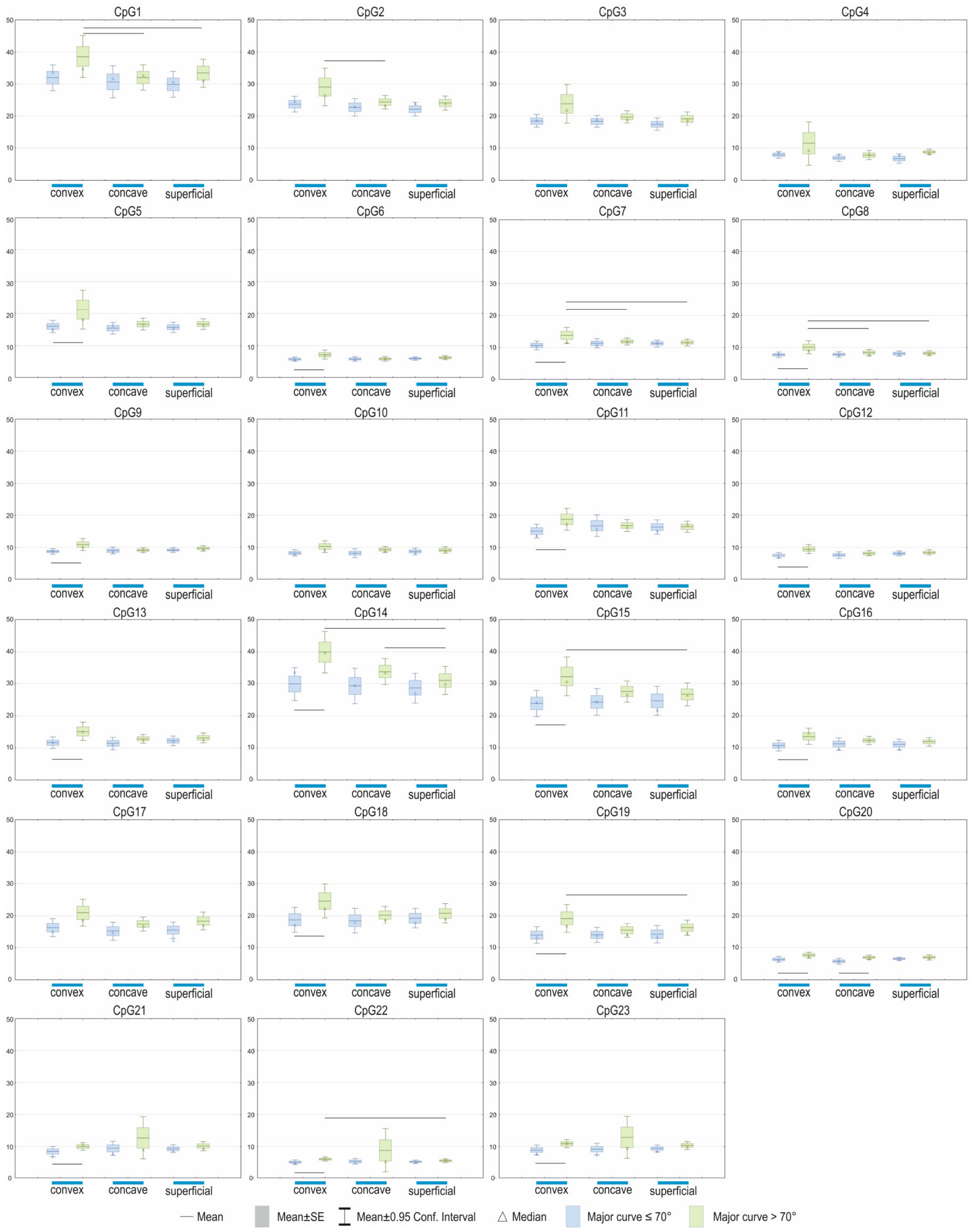

Figure S4. DNA methylation level within *LBX1* forward strand promoter region in deep paravertebral muscles and superficial muscles. Upper solid horizontal lines represent significant differences between muscles in case of patients, and lower lines show significant differences in subgroups divided according to major curve values.
